# Gedatolisib shows superior potency and efficacy versus single-node PI3K/AKT/mTOR inhibitors in breast cancer models

**DOI:** 10.1038/s41523-024-00648-0

**Published:** 2024-06-05

**Authors:** Stefano Rossetti, Aaron Broege, Adrish Sen, Salmaan Khan, Ian MacNeil, Jhomary Molden, Ross Kopher, Stephen Schulz, Lance Laing

**Affiliations:** Celcuity, Inc. 16305 36th Ave N, Suite 100, Minneapolis, MN 55446 USA

**Keywords:** Targeted therapies, Cancer metabolism

## Abstract

The PI3K, AKT, and mTOR (PAM) pathway is frequently dysregulated in breast cancer (BC) to accommodate high catabolic and anabolic activities driving tumor growth. Current therapeutic options for patients with hormone receptor (HR) + / HER2- advanced BC (ABC) include PAM inhibitors that selectively inhibit only one PAM pathway node, which can lead to drug resistance as cells rapidly adapt to maintain viability. We hypothesized that gedatolisib, which potently inhibits all Class I PI3K isoforms, as well as mTORC1 and mTORC2, may be more effective in BC cells than single-node PAM inhibitors by limiting adaptive resistances. By using multiple functional assays, a panel of BC cell lines was evaluated for their sensitivity to four different PAM inhibitors: gedatolisib (pan-PI3K/mTOR inhibitor), alpelisib (PI3Kα inhibitor), capivasertib (AKT inhibitor), and everolimus (mTORC1 inhibitor). Gedatolisib exhibited more potent and efficacious anti-proliferative and cytotoxic effects regardless of the PAM pathway mutational status of the cell lines compared to the single-node PAM inhibitors. The higher efficacy of gedatolisib was confirmed in three-dimensional culture and in BC PDX models. Mechanistically, gedatolisib decreased cell survival, DNA replication, cell migration and invasion, protein synthesis, glucose consumption, lactate production, and oxygen consumption more effectively than the other PAM inhibitors tested. These results indicate that inhibition of multiple PAM pathway nodes by a pan-PI3K/mTOR inhibitor like gedatolisib may be more effective at inducing anti-tumor activity than single-node PAM inhibitors. A global Phase 3 study is currently evaluating gedatolisib plus fulvestrant with and without palbociclib in patients with HR+/HER2− ABC.

## Introduction

**Fig. 1:**
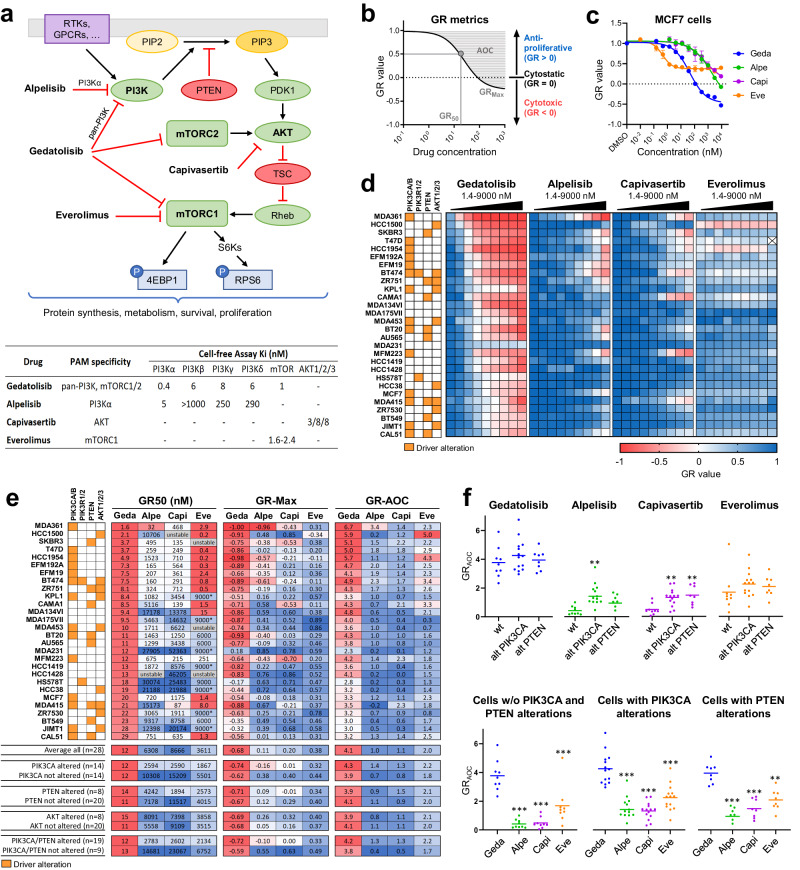
Analysis of PAM inhibitors response in BC cell lines using growth rate metrics. **a** Gedatolisib, alpelisib, capivasertib, and everolimus target PAM pathway nodes (top) with different specificity and affinity (bottom). **b** Anti-proliferative effects (GR value = 0–1), cytotoxic effects (GR < 0), potency (GR_50_), and efficacy (GR_Max_) of a drug can be evaluated using GR metrics. GR_AOC_ (area over the curve) captures both efficacy and potency. Lower GR_50_ indicates higher potency; higher GR_Max_ indicates higher efficacy; higher GR_AOC_ indicates higher potency and efficacy. **c** An example of GR metrics calculated from RTGlo MT values measured before and after PAM inhibitor treatment for 72 h is shown for the MCF7 cell line. Data represent mean ± SD (*n* = 2 biologically independent samples). **d** Heatmap showing GR values in response to 72-h treatment with increasing PAM inhibitor concentrations in 28 BC cell lines with various PAM pathway mutational status. See Supplementary Data 1 for values. **e** GR_50_, GR_Max_, and GR_AOC_ show that gedatolisib is more potent and efficacious than the other PAM inhibitors tested in most BC cell lines. Average values in subpopulations with or without altered PAM pathway genes are shown. * = Max concentration tested, GR_50_ not reached; Unstable = poor DRC fitting prevented reliable GR_50_ calculation. **f** GR_AOC_ analysis comparing potency and efficacy of gedatolisib, alpelisib, capivasertib, and everolimus in cell lines with driver genetic alterations in *PIK3CA* or *PTEN* (alt) and cell lines without driver genetic alterations in both *PIK3CA* and *PTEN* (wt). ** = *p* < 0.01, *** = *p* < 0.001 by Brown–Forsythe and Welch one-way ANOVA (top) or one-way ANOVA with Dunnet’s multiple comparisons (bottom). GR growth rate, geda gedatolisib, alpe alpelisib, capi capivasertib, eve everolimus, w/o without, wt wild type (i.e., no driver alterations), alt altered.

The PI3K, AKT, and mTOR (PAM) signaling pathway translates extracellular signals into specific cellular functions and thereby controls many aspects of cell physiology, including metabolic homeostasis, protein synthesis, cell survival, and proliferation^1,2^. Extracellular signals (e.g., growth factors, hormones, extracellular matrix components, nutrients) activate the PAM pathway through multiple membrane receptors, such as receptor tyrosine kinases (RTKs), G protein-coupled receptors (GPCRs), and integrins. Signal transduction through these receptors leads to PI3K-mediated conversion of phosphatidylinositol (4,5)-bisphosphate (PIP2) into phosphatidylinositol (3,4,5)-trisphosphate (PIP3), which in turn leads to activation of the PAM pathway (Fig. 1a)^1,2^. One of the main downstream effects of activated AKT is increased mTORC1 activity, which promotes anabolic processes (e.g., protein synthesis) required for cell growth and proliferation^3^. Other AKT effectors include GSK3 and FOXO, which play a key role in controlling cell metabolism, cell cycle, and cell survival, among other functions^1^. The conversion of PIP3 into PIP2 by the PTEN phosphatase, by counteracting PI3K activity, is one of the major termination mechanisms of PAM pathway signaling^1^.

Dysregulated PAM signaling is a common feature of a majority of tumors and is frequently associated with genetic alterations of key PAM pathway genes^2^. According to cBioPortal analysis of published cancer genomics datasets such as The Cancer Genome Atlas^4^, breast cancer is frequently characterized by genetic alterations of *PIK3CA* (~35–45%, mostly activating mutations) and *PTEN* (~6–10%, mostly inactivating mutations or deletions). Other studies confirmed the high prevalence of *PI3KCA* mutations (>30%) and reported PTEN loss in >30% BC specimens^5^. Mutations in other PAM pathway genes, dysregulation of interconnected pathways (e.g., RTKs), or epigenetic mechanisms may also affect PAM pathway activity in the absence of canonical *PIK3CA/PTEN* genetic alterations^6–8^.

Increased activation of PAM signaling induces many metabolic adaptations (e.g., increased glycolytic activity, Warburg effect) required for cancer cell growth and proliferation^9^. Due to the heavy reliance of tumor cells on the cellular functions controlled by PAM signaling (e.g., increased glycolysis), targeting this pathway was identified as a promising strategy for cancer treatment soon after the pathway was discovered^2,10^. Currently, three PAM inhibitors have received FDA approval for the treatment of HR+/HER2− advanced breast cancer (ABC) in combination with endocrine therapy: alpelisib (PI3Kα inhibitor), capivasertib (AKT inhibitor), and everolimus (mTORC1 inhibitor).

Non-clinical studies have provided important insights into the complexity and redundancy of the PAM pathway and have identified several feedback loops and compensatory mechanisms that can limit the therapeutic effect of more narrowly targeted PAM inhibitors, even when combined with other therapies^11^. Dual PI3K/mTOR inhibitors, by targeting multiple nodes of the PAM pathway, can overcome adaptive resistance mechanisms. While they are expected to be more efficacious than inhibitors targeting single PAM pathway nodes, clinical development of many dual PIK3/mTOR inhibitors, such as dactolisib^12^, was halted due to toxicity-related concerns.

Gedatolisib is an ATP-competitive and reversible dual PI3K/mTOR inhibitor^13,14^. Other dual PI3K/mTOR inhibitors show uneven targeting of PAM pathway nodes (e.g., dactolisib is less potent against PI3Kβ; apitolisib and samotolisib are less potent against mTOR), which may lead to reduced efficacy. In contrast, gedatolisib demonstrated similar nanomolar potency against all class I PI3K isoforms, as well as mTORC1 and mTORC2^13,14^ (Fig. 1a). Moreover, several clinical trials (Supplementary Table 1) have shown that gedatolisib is well tolerated in cancer patients. Results from early clinical studies in multiple tumor indications showed that therapy with gedatolisib produced promising preliminary efficacy with fewer patients experiencing class-associated side effects, such as hyperglycemia and gastrointestinal and skin toxicities when compared to published data for other PAM inhibitors^15–22^. Gedatolisib is currently being evaluated in combination with fulvestrant, with and without palbociclib, in patients with HR+/HER2− advanced breast cancer in a global Phase 3 clinical trial (VIKTORIA-1, NCT05501886).

In this study, we tested the pan-PI3K/mTOR inhibitor, gedatolisib, and node-selective inhibitors for PI3Kα (alpelisib), AKT (capivasertib) and mTORC1 (everolimus) to compare the functional effect of inhibiting single versus multiple PAM pathway nodes in a panel of breast cancer cell lines. Given the myriad adaptive cell responses that can maintain PAM function when a PAM pathway node is inhibited, assessment of antagonist activity required the use of objective metrics to characterize functional aspects of the pathway signaling. By employing multiple functional analyses, we demonstrate that inhibition of single PAM pathway nodes is less effective than pan-PI3K/mTOR inhibition at controlling PAM pathway activity and downstream cellular functions, such as protein synthesis, metabolism, cell cycle progression, survival, and proliferation in BC cells with mutant or wild type PAM pathway status. Consequently, multi-node PAM pathway inhibition results in more potent and efficacious anti-proliferative and cytotoxic effects on BC cells than single node inhibition.

## Results

### Analysis of PAM inhibitors response in BC cell lines using growth rate metrics and cell viability assays

The effect of single-node versus multi-node PAM inhibitors was first evaluated in a panel of 28 BC cell lines with and without PAM pathway alterations (Supplementary Table 2) by growth rate (GR) metrics analysis. Based on cell viability measurements taken before and after drug treatment, this analysis allows identification of cytostatic and cytotoxic effects (Fig. 1b) independent of cell doubling time^23^. Gedatolisib exerted potent, dose-dependent anti-proliferative and cytotoxic effects (Fig. 1c, d), with average GR_50_ = 12 nM and GR_Max_ = −0.68 (Fig. 1e). On average, gedatolisib was more potent and efficacious than single-node PAM inhibitors in all cell lines’ subpopulations analyzed, regardless of *PIK3CA* or *PTEN* mutational status (Fig. 1e and Supplementary Table 3). In cell lines with altered *PIK3CA* or *PTEN*, gedatolisib GR_50_ (12 nM) was at least 100-fold lower than the GR_50_ of alpelisib, capivasertib, and everolimus (2783, 2602, and 2134 nM, respectively) (Fig. 1e). In these cell lines, gedatolisib average GR_Max_ was −0.72 compared to −0.10, 0.00, and 0.33 for alpelisib, capivasertib, and everolimus, respectively (Fig. 1e), indicating that gedatolisib induced a large cytotoxic effect in most cell lines, while the single-node PAM inhibitors induced modest or no cytotoxic effects.

To test whether the PAM inhibitors had different effects related to PAM pathway mutational status, we compared their GR_AOC_ (which captures both efficacy and potency) in cell lines with or without *PIK3CA* and *PTEN* driver alterations (Fig. 1f). Gedatolisib GR_AOC_ was nearly identical between wild type or altered *PIK3CA*/*PTEN* cells lines, indicating that gedatolisib potency and efficacy was not influenced by *PIK3CA* or *PTEN* status. In contrast, alpelisib and capivasertib showed significantly higher GR_AOC_ in cell lines with altered *PIK3CA* and/or *PTEN* relative to those lacking these alterations (Fig. 1f). On average, the GR_AOC_ for gedatolisib was at least 70% higher (i.e., more potent and efficacious) than alpelisib, capivasertib, and everolimus regardless of the *PIK3CA* or *PTEN* mutational status (Fig. 1f). Gedatolisib was also more potent and efficacious than alpelisib, capivasertib, and everolimus regardless of ER/HER2 status (Supplementary Fig. 1) or sensitivity to the CDK4/6 inhibitor, palbociclib (Supplementary Fig. 2).

The same panel of BC cell lines was also evaluated for cell viability and cell death by using classical RT-Glo MT and Sytox assays at the end of a 72-hour treatment. Consistent with the GR metrics analysis, endpoint analysis of cell viability showed that gedatolisib was, on average, more potent and efficacious than single-node PAM inhibitors in all cell lines’ subpopulations analyzed (Supplementary Fig. 3a, b). Parallel staining with Sytox green also showed that gedatolisib induced cell death in a dose-dependent manner in most cell lines, regardless of *PIK3CA* or *PTEN* mutational status (Supplementary Fig. 3c). Gedatolisib-induced cell death was associated with increased levels of cleaved caspase 3 (Supplementary Data 4), indicating induction of apoptosis as previously described both in vitro and in vivo^13^. On average, gedatolisib induced greater cell death than alpelisib, capivasertib, and everolimus in both cell lines with altered *PIK3CA* or *PTEN* (maximal cell death induced = 53% versus 21%, 14%, and 4%, respectively) and cell lines with wild type *PIK3CA* and *PTEN* (maximal cell death induced = 48% versus 7%, 4%, and 6%, respectively) (Supplementary Data 3).

Overall, the GR metrics and cell viability results demonstrated that multi-node inhibition by gedatolisib exerted greater anti-proliferative and cytotoxic effects than single-node inhibition in BC cells, regardless of *PIK3CA* or *PTEN* status. Based on these results, we set out to test whether the multi-node and single-node PAM inhibitors also exerted a different effect on PAM pathway activity and PAM-controlled cellular functions.

### Analysis of PAM pathway activity in response to PAM inhibitors

We first quantified PI3K-related cell signaling pathway activity by exposing live tumor cells derived from four BC patients’ tumor tissue samples (Supplementary Fig. 4a) to PAM inhibitors and evaluating them using the CELsignia PI3K Signaling Pathway test. PI3K signaling was stimulated with a GPCR agonist (125 nM LPA) for 4 h and drug response was assessed by measuring the inhibition of agonist-induced impedance changes. As shown in Supplementary Fig. 4b, gedatolisib completely abrogated LPA-induced PI3K signaling (IC_50_ < 50 nM). Neither alpelisib nor capivasertib reduced total cell activity by 50% at concentrations up to 1000 nM.

**Fig. 2:**
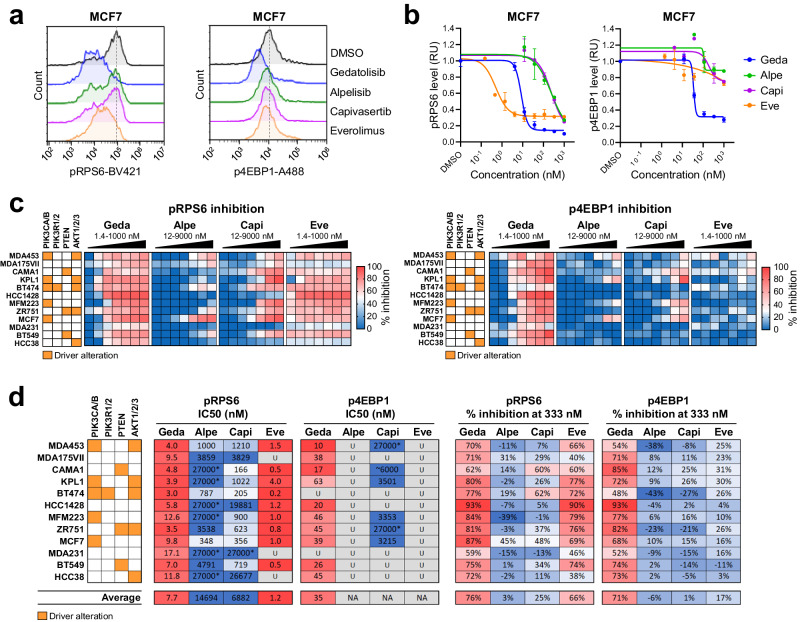
Analysis of PAM pathway activity in response to PAM inhibitors. **a** Example of flow cytometric analysis showing p4EBP1(T36/T45) and pRPS6(S235/S236) levels after 48 h treatment with 333 nM PAM inhibitors in MCF7 cells. **b** pRPS6 and p4EBP1 median fluorescence intensities normalized to DMSO-treated cells (set as 1) were used to plot PAM inhibitors DRCs as shown here for MCF7. Data represent mean ± SD (*n* = 2 biologically independent samples). **c** Heatmaps showing pRPS6 and p4EBP1 levels in response to increasing PAM inhibitor concentrations in a panel of BC cell lines. The % inhibition was calculated from median fluorescence intensity and is relative to DMSO-treated cells. See Supplementary Data 5 and 6 for values. **d** Comparison of PAM inhibitors potency (absolute IC_50_) and efficacy (% inhibition at 333 nM) in inhibiting pRPS6 and p4EBP1. * Max concentration tested; IC_50_ not reached; U unstable DRC prevented reliable IC_50_ calculation, BV421 brilliant violet 421, A488 Alexa Fluor 488, geda gedatolisib, alpe alpelisib, capi capivasertib, eve everolimus, RU relative units.

Next, we evaluated PAM pathway inhibition in response to each PAM inhibitor by measuring the phosphorylation status of downstream effectors, such as 4EBP1 and RPS6. Representative data are shown in Fig. 2a, b, while a summary of pRSP6 and p4EBP1 inhibition in 12 BC cell lines is shown in Fig. 2c. On average, gedatolisib decreased pRPS6 and p4EBP1 levels by 76% and 71% at 333 nM, respectively, for all cell lines tested while average IC_50_ levels were 8 nM and 35 nM, respectively (Fig. 2d). Alpelisib, capivasertib, and everolimus decreased pRPS6 levels 3%, 25% and 66% at 333 nM, respectively. Everolimus decreased p4EBP1 levels 17%, while alpelisib and capivasertib had almost no effect on p4EBP1 (Fig. 2d). Average IC_50_ levels for alpelisib, capivasertib, and everolimus for pRPS6 were 14,694 nM, 6882 nM, and 1.2 nM, respectively; average IC_50_ values for p4EBP1 could not be determined for any of these drugs due to lack of potency (Fig. 2d). Interestingly, the baseline levels of p4EBP1 in DMSO-treated cells did not correlate with gedatolisib GR metrics (data not shown), suggesting that baseline PAM activity assessed by p4EBP1 is not a predictive biomarker of gedatolisib efficacy in vitro.

These complementary approaches showed that multi-node PAM pathway inhibition decreased PAM pathway activity more effectively than single node inhibition. We next employed a series of functional analyses to test whether the differential effect of the various PAM inhibitors on PAM pathway activity translated into a differential effect on PAM-controlled functions critical for cancer development and progression: protein synthesis, cell cycle, and DNA replication, migration and invasion, and metabolic functions.

### Analysis of protein synthesis in response to PAM inhibitors

The PAM pathway, through mTORC1 and its effectors 4EBP1 and S6Ks, plays a critical role in controlling protein synthesis^24^. To assess the effect of PAM inhibitors on protein synthesis, four BC cell lines with different *PIK3CA* and *PTEN* mutational status were treated with increasing concentrations of gedatolisib, alpelisib, capivasertib, or everolimus for approximately 20 h and analyzed by flow cytometry for protein synthesis (assessed by OPP incorporation) as well as for p4EBP1 and pRPS6 (used as a marker of S6Ks activity).

**Fig. 3:**
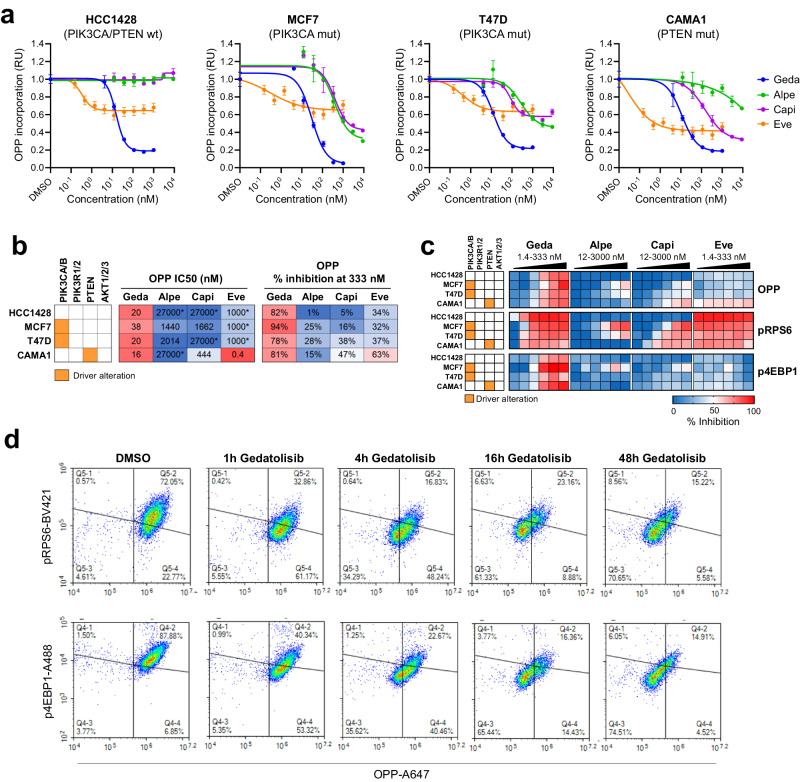
Analysis of protein synthesis in response to PAM inhibitors. **a** Analysis of OPP incorporation in BC cells lines treated with increasing concentrations of PAM inhibitors for approximately 20 h. OPP median fluorescent intensity was normalized to DMSO-treated cells. Data represent mean ± standard deviation (*n* = 2 biologically independent samples). **b** Comparison of PAM inhibitors potency (absolute IC_50_) and efficacy (% OPP inhibition at 333 nM) in inhibiting protein synthesis. * Maximum concentration tested; IC_50_ not reached. **c** Concomitant analysis of OPP incorporation, pRPS6, and p4EBP1 by flow cytometry in cells treated with increasing PAM inhibitor concentrations for approximately 20 h. The % inhibition was calculated from median fluorescence intensity and is relative to DMSO-treated cells. See Supplementary Data 7 for values. **d** Time course analysis of OPP incorporation, pRPS6 (top), and p4EBP1 (bottom) in T47D cells treated with 333 nM gedatolisib. wt wild type, mut mutant, OPP O-propargyl-puromycin, BV421 Brilliant Violet 421, A488 Alexa Fluor 488, A647 Alexa Fluor 647, geda gedatolisib, alpe alpelisib, capi capivasertib, eve everolimus, RU relative units.

Gedatolisib effectively reduced OPP incorporation in all four cell lines tested with IC_50_ values < 40 nM (Fig. 3a, b). At 333 nM, OPP incorporation was inhibited between 78-94% (Fig. 3b). Alpelisib, capivasertib and everolimus were less potent than gedatolisib, except for one cell line (CAMA1) where everolimus was more potent than gedatolisib. At 333 nM, alpelisib, capivasertib, and everolimus were less efficacious than gedatolisib at inhibiting OPP incorporation across all cell lines (Fig. 3b). Consistent with the OPP results, alpelisib, capivasertib, and everolimus were less effective than gedatolisib in reducing p4EBP1 levels; alpelisib and capivasertib were also less effective in reducing pRPS6 levels (Fig. 3c). Time course experiments in T47D cells were performed to obtain additional insight about the effect of gedatolisib on OPP incorporation, pRPS6 and p4EBP1. In these cells, gedatolisib at 333 nM inhibited OPP incorporation within 4 h, and the effect started to plateau at approximately 16 h. Both pRPS6 and p4EBP1 levels were reduced by gedatolisib (333 nM) after 1 h treatment and remained low up to 48 h (Fig. 3d).

These results showed that the differential inhibition of the PAM pathway activity induced by gedatolisib and the other PAM inhibitors also resulted in a correlative inhibition of protein synthesis, one of the major cellular functions directly controlled by the PAM pathway.

### Analysis of cell cycle and DNA replication in response to PAM inhibitors

**Fig. 4:**
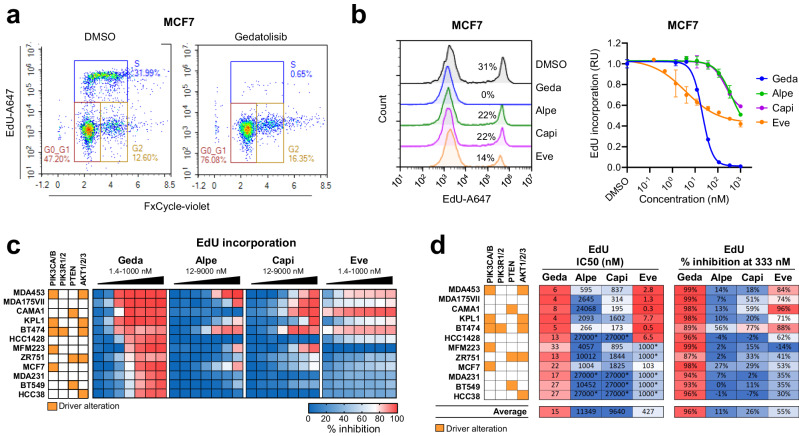
Analysis of cell cycle and DNA replication in response to PAM inhibitors. **a** Example of cell cycle analysis by flow cytometry in MCF7 treated with or without gedatolisib (333 nM) for 48 h. **b** Example of EdU analysis by flow cytometry in MCF7 cells treated for 48 h treatment with or without 333 nM PAM inhibitors (left). The % of EdU + cells normalized to DMSO-treated cells (set as 1) was used to plot PAM inhibitors DRCs, as shown here for MCF7. Data represent the mean ± SD, *n* = 2 biologically independent samples (right). **c** Heatmap showing inhibition of EdU incorporation in response to increasing PAM inhibitors concentrations in a panel of 12 BC cell lines. See Supplementary Data 8 for values. **d** Comparison of PAM inhibitors potency (absolute IC_50_) and efficacy (% inhibition at 333 nM) in inhibiting EdU incorporation. *Maximum concentration tested; IC_50_ not reached. EdU 5-ethynyl-2′-deoxyuridine, A647 Alexa Fluor 647, geda gedatolisib, alpe alpelisib, capi capivasertib, eve everolimus, RU relative units.

Another important function of the PAM pathway is the control of cell cycle and cell proliferation^1^. To test the effect of the PAM inhibitors on the cell cycle and DNA replication, we used the EdU incorporation assay. A panel of 12 BC cell lines with various PAM pathway mutational status were treated with increasing concentrations of gedatolisib, alpelisib, capivasertib, or everolimus for approximately 48 h. During the last 2 h of treatment, cells were incubated with EdU, a thymidine analog that is incorporated into newly synthesized DNA during the S phase of the cell cycle (as shown in Fig. 4a). Representative EdU incorporation data in response to PAM inhibitors in MCF7 cells are shown in Fig. 4b, and the percentage inhibition of EdU incorporation for all 12 cell lines tested is summarized in Fig. 4c. Gedatolisib completely or almost completely blocked EdU incorporation in all cell lines with an average IC_50_ of 15 nM (Fig. 4d). On average, alpelisib, capivasertib, and everolimus were less potent (IC_50_ = 11,349 nM, 9,640 nM, and 427 nM, respectively) than gedatolisib in all cell lines. However, everolimus showed similar potency to gedatolisib in 6 of the 12 cell lines. On average, gedatolisib (333 nM) induced 96% inhibition of EdU incorporation, compared to 11%, 26%, and 55% for alpelisib, capivasertib, and everolimus, respectively (Fig. 4d).

Based on these findings, gedatolisib demonstrated better efficacy than the other PAM inhibitors at blocking cell cycle and DNA replication. The greater inhibition of cell cycle and DNA replication, along with the greater induction of cell death observed by Sytox staining (Supplementary Fig. 3c), provide a mechanistic explanation for the greater efficacy of gedatolisib versus the other PAM inhibitors observed by GR metrics analyses.

### Analysis of migration and invasion in response to PAM inhibitors

The PAM pathway is involved, directly or through interaction with other signaling pathways, in cytoskeleton remodeling, epithelial mesenchymal transition (EMT), cell migration, and invasion^25^. The effects of multi-node and single-node PAM inhibitors on migration and invasion were compared by using transwell assays in MDA-231, a BC cell line with well characterized migratory and invasive properties. As shown in Supplementary Fig. 5a and Supplementary Data 9, gedatolisib inhibited MDA-231 migration more effectively than alpelisib, capivasertib and everolimus (88% versus 70%, 20%, and 39%, respectively, at the maximum concentration tested). Similar results were also observed in the invasion assay, where gedatolisib (100 nM) inhibited MDA-231 invasion through Matrigel by 64% versus 39% for alpelisib (1000 nM), and no inhibition for capivasertib and everolimus (Supplementary Fig. 5b and Supplementary Data 10). Both everolimus and capivasertib induced, on average, an increase in cell invasion; however, due to the high variance specifically observed with these two PAM inhibitors (see Supplementary Data 10), this increase was mostly not significant.

### Analysis of metabolic functions in response to PAM inhibitors

Increased activation of the PAM pathway plays a key role in driving metabolic adaptations required by cancer cells to sustain biomass and energy production, cell proliferation, and cell movement. Such adaptations include enhanced glucose uptake and glycolysis, with a consequent increase in lactate production^9,26^. Therefore, it is likely that the different effects of multi-node and single-node PAM inhibitors on cellular functions like cell proliferation and migration are linked to different effects on cancer cells’ metabolism. To address this question, we tested the impact of gedatolisib, alpelisib, capivasertib, and everolimus on key metabolic activities.

**Fig. 5:**
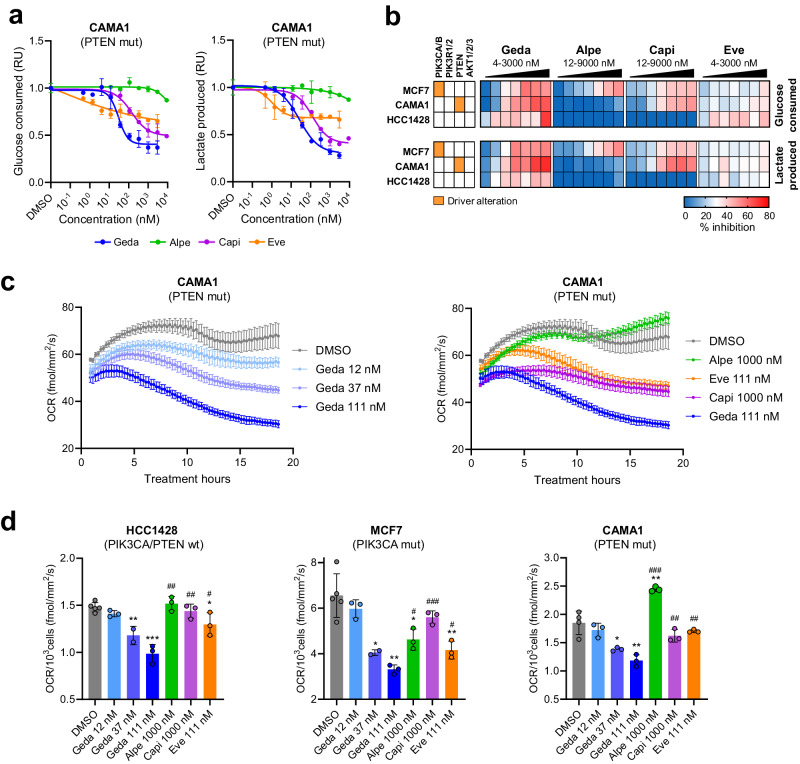
Analysis of metabolic functions in response to PAM inhibitors. **a** Example of DRCs showing the effect of a 24-hour PAM inhibitor treatment on glucose consumption and lactate production in CAMA1 cells. Data are normalized to cell number and shown relative to DMSO-treated cells (set as 1). Data represent mean ± SD, *n* = 3 biologically independent samples. **b** Heat maps summarizing cell number-normalized glucose consumption and lactate production in response to PAM inhibitors in a panel of BC cell lines. See Supplementary Data 11 for values. **c**, **d** Resipher analysis of OCR in BC cell lines treated with PAM inhibitors for approximately 18 h. The graphs in **c** show time-course analysis of OCR in CAMA1 as an example (MCF7 and HCC1428 are shown in Supplementary Fig. 6). The graphs in **(d)** show OCR normalized to the number of cells at the end of treatment. Data represent mean ± SEM (*n* = 2–5 biologically independent samples). Two-tailed, unpaired *t*-test, *p < 0.05 **p < 0.01 ***p < 0.001 vs DSMO; ^#^*p* < 0.05 ^##^*p* < 0.01 ^###^ *p* < 0.001 vs 111 nM gedatolisib. See Supplementary Data 12 for values. RU relative units, OCR oxygen consumption rate, wt wild type, mut mutant, geda gedatolisib, alpe alpelisib, capi capivasertib, eve everolimus.

First, we compared the effects of gedatolisib and single-node PAM inhibitors on glucose consumption and lactate production in three BC cells lines with various *PIK3CA/PTEN* mutational status. After treatment for 20–24 h with increasing drug concentrations, the conditioned medium was analyzed for changes in glucose and lactate levels relative to unconditioned medium. As shown in Fig. 5a, b, gedatolisib reduced glucose consumption and lactate production in a dose-dependent manner, and these changes were independent of cell number. On average, gedatolisib inhibited glucose consumption and lactate production up to 55–60% (see Supplementary Data 11) compared to <40% for alpelisib, capivasertib and everolimus.

Next, we tested the effects of the PAM inhibitors on oxygen consumption rate (OCR). Cells were treated with gedatolisib, alpelisib, capivasertib, or everolimus overnight, and O_2_ levels in the medium were monitored in real time using a Resipher platform to assess OCR (Fig. 5c and Supplementary Fig. 6). Gedatolisib decreased OCR in a time- and dose-dependent fashion up to 33% in HCC1428 (*PTEN/PIK3CA* wild type), 49% in MCF7 (*PIK3CA* mutant) and 36% in CAMA1 (*PTEN* mutant) (Fig. 5d and Supplementary Data 12). In all cell contexts, gedatolisib reduced OCR more effectively than the three single-node PAM inhibitors when tested at concentrations shown to inhibit cell growth of sensitive BC cancer cell lines (Fig. 5d and Supplementary Data 12).

Overall, our functional analyses showed that the greater inhibition of PAM pathway activity induced by gedatolisib versus single-node PAM inhibitors also resulted in greater inhibition of PAM-controlled functions, such as protein synthesis, cell cycle and DNA replication, cell migration and invasion, and metabolic activities required for cancer cell survival and proliferation.

### Analysis of PAM inhibitors in three-dimensional culture

Three-dimensional (3D) in vitro cell culture systems are suggested to provide some in vivo, physiologically relevant perspectives on therapeutic activity and are increasingly used for drug testing^27^. To confirm the effect of gedatolisib, alpelisib, capivasertib, and everolimus observed in assays using standard two-dimensional (2D) cultures, MCF7 (*PIK3CA* mutant) and HCC1428 (*PIK3CA* wild type) cells were grown in 3D culture on basement membrane extracts (BME). Since these cell lines had also been tested in 2D culture, the effects of the 2D and 3D culture conditions on PAM inhibitors response can be compared.

Gedatolisib and the single-node PAM inhibitors were first tested to assess their ability to inhibit growth of 3D cancer cell spheroids. Single cells were seeded on BME and treated with each PAM inhibitor approximately 20 h post seeding. As shown in Supplementary Fig. 7, six days after treatment, gedatolisib (333 nM) inhibited 3D growth of HCC1428 and MCF7 cells more effectively than alpelisib, capivasertib, and everolimus.

**Fig. 6:**
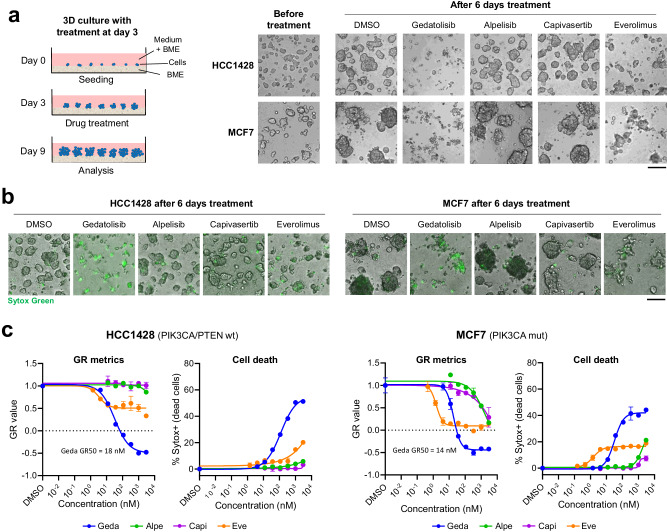
Analysis of PAM inhibitors in three-dimensional culture. **a** HCC1428 and MCF7 spheroids were grown in 3D culture on basement membrane extract (BME) for 3 days before treatment with PAM inhibitors for 6 days (left). Micrographs taken after 6 days of treatment show that 333 nM gedatolisib inhibited 3D growth and induced spheroid regression more effectively than the other PAM inhibitors tested at the same concentration (right). Scale bar = 200 µm. **b** Sytox Green staining of 3D cultures treated for 6 days showed that spheroid regression induced by gedatolisib was marked by extensive cell death. Scale bar = 200 µm. **c** GR metrics and cell death analysis by Sytox Green show that gedatolisib has more potent and efficacious anti-proliferative and cytotoxic effects than the other PAM inhibitors tested in HCC1428 and MCF7 3D cultures treated for 6 days. Data represent mean ± SD (*n* = 2 biologically independent samples). 3D three-dimensional, BME basement membrane extract, GR growth rate, wt wild type, mut mutant.

The activity of each PAM inhibitor was then tested on MCF7 and HCC1428 3D spheroids grown for three days before treatment. These conditions allowed us to assess whether the PAM inhibitors not only reduced spheroid growth but could also induce spheroid regression. Treatment with gedatolisib (333 nM) for 6 days clearly reduced spheroid size (Fig. 6a) by inducing cell death (assessed by Sytox staining; Fig. 6b). Treatment with 333 nM alpelisib or capivasertib had modest anti-proliferative effects on spheroid growth relative to DMSO controls, while treatment with 333 nM everolimus blocked spheroid growth but did not induce spheroid regression to the extent gedatolisib did (Fig. 6a, b). These results were confirmed by dose response GR metrics and cell death analyses, where gedatolisib demonstrated greater anti-proliferative and cytotoxic effects relative to the three single-node PAM inhibitors (Fig. 6c).

These experiments confirmed the greater efficacy of multi node versus single node PAM inhibition in a 3D context that mimics key features of the tumor microenvironment. To further validate these results, we compared the in vivo efficacy of the various PAM inhibitors by using mini-patient derived xenograft (mini-PDX) BC models.

### Analysis of PAM inhibitors in BC mini-PDX mouse models

**Fig. 7:**
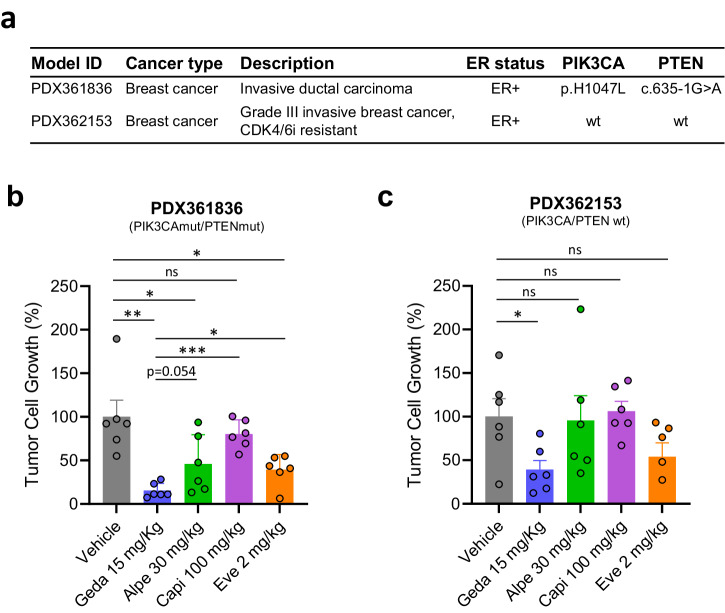
Analysis of PAM inhibitors in BC mini-PDX mouse models. **a** Characteristics of the mini-PDX models tested. **b**, **c** PAM inhibitors efficacy in mini-PDX models. Encapsulated cells were injected subcutaneously in the flank of female BALB/c nude mice and animals were treated with the indicated PAM inhibitors for 7 days. Gedatolisib: i.v. Q4D; alpelisib: p.o. QD; capivasertib: p.o. BID 4 days on/3 days off; everolimus: p.o. QD. Data represents mean ± SEM (*n* = 6 mini-pDX capsules) ns not significant, * *p* < 0.05, ** *p* < 0.01; *** *p* < 0.001. wt wild type, mut mutant, geda gedatolisib, alpe alpelisib, capi capivasertib, eve everolimus.

The mini-PDX approach is a faster alternative to classical PDX models, without a loss in predictive power for drug response^28^. The in vivo efficacy of the PAM inhibitors was tested in two different mini-PDX BC models, one with *PIK3CA*/*PTEN* mutations and one lacking *PIK3CA*/*PTEN* alterations (Fig. 7a).

In the *PIK3CA*/*PTEN* mutant model (PDX361836, Fig. 7b), gedatolisib, alpelisib, and everolimus induced significant tumor cell growth inhibition (TCGI) relative to vehicle (85%, 55%, 60% with *p* = 0.001, *p* = 0.043, *p* = 0.014, respectively), while capivasertib induced modest, non-significant TCGI (20%, *p* = 0.35). The TCGI induced by gedatolisib was higher than the TCGI induced by other PAM inhibitors (alpelisib, *p* = 0.054; capivasertib, *p* < 0.001; everolimus, *p* = 0.013). In the *PIK3CA*/*PTEN* wild type model (PDX362153, Fig. 7c), gedatolisib induced statistically significant TCGI (>60%, *p* = 0.025) relative to vehicle, while alpelisib, capivasertib, and everolimus failed to induce statistically significant TCGI. Of note, the PDX362153 wild-type model was derived from a BC patient who had received prior treatment with a CDK4/6 inhibitor, suggesting that gedatolisib can be effective in this BC patient subpopulation. In both models, the PAM inhibitors did not induce significant changes in mouse body weight relative to vehicle controls, indicating lack of toxicity (Supplementary Fig. 8). These in vivo studies confirmed the in vitro findings and showed that gedatolisib was the only PAM inhibitor inducing significant TCGI in both wild type and mutant *PIK3CA/PTEN* PDX models.

## Discussion

Excessive glucose consumption by tumors and the promotion of carbon molecules into anabolic processes have described tumor metabolism for nearly a century. The key role of the PAM pathway in controlling anabolism and catabolism in cancer cells with or without PAM pathway mutations makes the PAM pathway an attractive therapeutic target. However, the clinical development of PAM inhibitors is challenging because resistance mechanisms can be induced when single PAM pathway nodes are inhibited. In addition, clinically significant adverse drug reactions, such as hyperglycemia, can occur when metabolic homeostasis is disrupted. These challenges proved insurmountable for many PAM inhibitors for which development was halted due to lack of efficacy, poor tolerability, or both^29^. Pan-PI3K/mTOR inhibitors have been hypothesized to be more effective than inhibitors targeting single PAM pathway nodes because they can induce more comprehensive PAM pathway inhibition^30,31^. Our study shows that gedatolisib, an equipotent pan-PI3K/mTOR inhibitor well tolerated in patients, is more effective at inhibiting PAM pathway functions than single-node PAM inhibitors such as alpelisib (PI3Kα), capivasertib (AKT) and everolimus (mTORC1), and induces greater growth-inhibitory and cytotoxic activity in BC cells with altered or wild type PAM pathway status.

Safety for administration of PAM inhibitors has been a concern. We note that gedatolisib’s average IC_50_ in BC cancer cells (approximately 40 nM) was much lower than the IC_50_ reported for normal cells, such as HEK293 (690 nM) and PBMC (IC_50_ not reached at micromolar concentrations)^32,33^. This observation suggests that gedatolisib could target cancer cells without adversely affecting normal cells, which is consistent with the manageable side effects observed in patients treated with gedatolisib.

Some PAM inhibitors can induce systemic hyperglycemia and hyperinsulinemia if they attain high concentrations in either the liver or pancreas, or both, before being systemically distributed. Goncalves et al. reviewed three steps for how PI3K pathway inhibitors can lead to sustained hyperglycemia in patients via a suppressed intracellular response to insulin: reduction in glucose uptake with glycolysis, increased glycogenolysis, and increased gluconeogenesis^34^. Multi-node PAM inhibitors like gedatolisib clearly reduce glucose consumption and glycolysis in cells more effectively than single-node PAM inhibitors. This leaves decreased glycogenolysis and gluconeogenesis as potential mechanisms for the reduction in a glucagon/insulin response. Clinical studies^20,22^ report that gedatolisib has a reduced rate of hyperglycemia in patients when compared to published data for other PAM inhibitors^16,17^. We hypothesize that gedatolisib may not affect pancreatic islet cells, hepatocyte potency, or drug uptake sufficiently to trigger glucagon release, glycogenolysis, and gluconeogenesis that would, in turn, lead to sustained systemic hyperglycemia and/or hyperinsulinemia. Additional studies will be needed to identify which, if any, of these mechanisms may be responsible for the differential effect of multi-node and single-node PAM inhibitors on glucose homeostasis.

To compare potency and efficacy of single-node and multi-node PAM inhibitors, we used different approaches, including classical endpoint cell viability assays and GR metrics analyses. These different approaches demonstrated that pan-PI3K/mTOR inhibition by gedatolisib generally exerted more potent and efficacious anti-proliferative/cytotoxic effects than single-node PAM inhibitors, regardless of the tumor cells’ PAM pathway mutational status. The results for each of the inhibitors are in line with previously published in vitro data, which showed similar IC_50_ levels and sensitivity in cancer cells treated with gedatolisib, alpelisib, or capivasertib under similar conditions^13,35,36^. Remarkably, the on-cell potencies of alpelisib and capivasertib were much lower than their cell-free potencies, whilst there was little difference between the potencies for gedatolisib. We also confirmed that everolimus can have high potency but low efficacy even in sensitive cell lines^37^, and that alpelisib and capivasertib are more efficacious in cell lines with altered *PIK3CA* and/or *PTEN* than wild type cell lines^35,36^.

A significant finding of this study is that gedatolisib was similarly effective in cell lines with or without genetic alterations of PAM pathway genes. The association between increased PAM pathway activity and PAM pathway mutations may explain why some PAM inhibitors are more effective in cell lines with mutated PAM pathway genes. However, cancer cells can have increased PAM pathway activity due to factors other than canonical *PIK3CA* and *PTEN* mutations^8^. Thus, BC patients without *PIK3CA* or *PTEN* mutations (>50% of BC patients) may have an increased activation of the PAM pathway that could be targeted more effectively by a multi-node PAM inhibitor like gedatolisib than a single-node PAM or mutant-specific inhibitor. Experiments showing that inhibition of all class I PI3K isoforms by copanlisib was less effective than panPI3K/mTOR inhibition by gedatolisib in cell lines with wild type *PIK3CA* and *PTEN* (Supplementary Fig. 9) suggest that inhibition of mTOR may be required for effective cell growth inhibition in this BC subpopulation. Interestingly, concomitant inhibition of PI3Kα and mTORC1 by alpelisib and everolimus was not as effective as gedatolisib in BC cell lines (Supplementary Fig. 10), indicating that comprehensive inhibition of all class I PI3K isoforms, mTORC1, and mTORC2 is critical for increased efficacy.

A likely explanation for gedatolisib higher potency and efficacy relative to single-node PAM inhibitors may lie in its ability to induce greater inhibition of the PAM signaling and PAM-controlled cell functions. Phospho-markers like pRPS6 and p4EBP1 have been extensively used to gauge PAM pathway activity in response to PAM inhibitors, both in non-clinical and clinical studies^38^. Gedatolisib induced a durable (48 h) and more pronounced decrease in both pRPS6 and p4EBP1 than the single-node PAM inhibitors, indicating more effective inhibition of PAM pathway activity. Remarkably, single node PAM inhibition had very modest effects on p4EBP1, even when it reduced pRPS6 (e.g., everolimus). This is consistent with published evidence that effective 4EBP1 dephosphorylation requires combined PI3K and mTORC1 inhibition^39^. Efficient inhibition of 4EBP1 phosphorylation has been suggested to be critical for suppression of cancer cell proliferation^40^, and could explain, at least in part, the superior efficacy of gedatolisib versus single-node PAM inhibitors.

To further understand the mechanisms underlying the different growth-inhibitory effects of multi-node and single-node PAM inhibitors, we employed a series of assays to analyze the functional output of PAM pathway inhibition.

First, the decrease of p4EBP1 and pRPS6 induced by gedatolisib was rapidly (<4 h) followed by a significant decrease in protein synthesis, which was more pronounced compared to the single-node PAM inhibitors. Cancer cells critically rely on increased protein synthesis to sustain their higher proliferation rate. In addition, cancer cells also hijack the translation machinery to promote the synthesis of proteins involved in tumor initiation, maintenance, and dissemination (e.g., cyclins, VEGF, BCL-xL, MMP3)^24,41^. Thus, effective inhibition of protein synthesis through comprehensive targeting of the PAM pathway is expected to have collateral impact on several cancer-driver functions.

Second, gedatolisib was more effective than single-node PAM inhibitors at inhibiting cell cycle progression through S-phase and inducing cell death. Inhibition of the PAM pathway is expected to impact multiple proteins involved in cell cycle control, e.g., p21 and p27 through the AKT-FOXO axis, cMyc through the AKT-GSK3 axis, and cyclin D1 through the mTORC1 effectors 4EBP1 and S6K1^1,42^. In addition, AKT exerts an anti-apoptotic action through various AKT effectors, such as FOXO transcription factors and BCL2 associated agonist of cell death (BAD)^6^. Consequently, gedatolisib’s comprehensive inhibition of the PAM pathway can reduce cell proliferation by inhibiting cell cycle progression and promote cell death by relieving AKT-controlled anti-apoptotic pathways.

Third, we found that gedatolisib decreased OCR, glucose consumption, and lactate production more effectively than single-node PAM inhibitors. These metabolic changes can have a direct impact on cancer cells by diminishing catabolic and anabolic activities required for cell growth and proliferation^9,26^. In addition, due to the key role of glycolysis in preventing apoptosis and fueling EMT^43,44^, effective inhibition of glucose metabolism could be linked to the induction of apoptosis and the decreased migration and invasion we observed after gedatolisib treatment. Perhaps more importantly, the reduced metabolic activities within the cancer cells can also affect the tumor microenvironment (TME). Low glucose levels, high lactate (with consequent decrease in pH), and hypoxia induced by tumor cells in the TME can impose critical metabolic restrictions for anti-tumor immune cells and promote immune suppressor cells^45^. Efficient inhibition of cancer cells’ glycolysis and OCR by gedatolisib could “normalize” the TME and consequently improve anti-tumor immune response. Consistent with this hypothesis, gedatolisib was reported to induce infiltration and activation of anti-tumor immune cells (e.g., CD8+ cytotoxic T-cells) in PyMT mouse mammary tumors^46^.

These functional analyses indicate that multi-node PAM inhibitors like gedatolisib are more effective than single-node PAM inhibitors at controlling key cellular functions induced by PAM pathway activation and required by cancer cells for energy production, molecule biosynthesis, survival, and cell cycle progression. This is likely to translate into more effective anti-proliferative/cytotoxic effects and, ultimately, into increased tumor growth inhibition.

Several mechanisms of adaptive resistance could explain the lower efficacy of single-node PAM inhibitors^11^. For instance, chronic PI3Kα antagonism can lead to increased PI3Kβ activity and vice versa^47^; AKT inhibition relieves feedback suppression of RTK expression and activity^48^ and activates SGK3 to maintain activated mTOR function^49^; mTORC1 inhibition, by relieving the mTORC1 negative feedback loops, can lead to the reactivation of PI3K-AKT signaling through stimulation of IRS1/2^1,50^ and reduction of PTEN translation via 4EBP1^51^. Equipotent inhibition of all PI3K isoforms, mTORC1, and mTORC2 is expected to prevent or counteract some of these resistance mechanisms. For instance, pan-PI3K inhibition could control the increased PI3Kβ activity induced by PI3Kα antagonism, while concomitant PI3K/mTORC1 inhibition could counteract PI3K reactivation through the mTORC1-IRS-PI3K or the mTORC1-4EBP1-PTEN feedback loops. Another resistance mechanism is the suppression of the insulin feedback loop, whereby systemic hyperglycemia and hyperinsulinemia induced by PI3K inhibition can reactivate the PAM pathway in cancer cells^52^. Gedatolisib induces a lower rate of hyperglycemia when compared to published data for single-node PAM inhibitors^16,17,20,22^. This would thus potentially avoid activation of an insulin signal that could reduce gedatolisib’s efficacy.

To treat BC, PAM inhibitors are currently approved for use in combination with various endocrine therapies. This strategy partially reflects the central role demonstrated in non-clinical studies for the linkage between estrogen receptor, cell cycling, and PAM pathways in cancer cells proliferation and adaptive drug resistance^53–55^. PAM inhibitors, including gedatolisib, may not be fully effective when used as single agents^31,56^. The non-clinical reports suggest that a PAM inhibitor’s effectiveness in patients with BC would be enhanced when combined with a selective estrogen receptor degrader alone or also in combination with a CDK4/6 inhibitor^57–59^. The optimal PAM inhibitor in this combination would also be one that prevents the adaptive resistance that occurs within the PAM pathway when only a single PAM node is inhibited.

The effect of combining gedatolisib with a SERD (fulvestrant), and a CDK4/6 inhibitor (palbociclib), alone or together, was evaluated in a previous study with the MCF7 BC xenograft model. While each drug as a single agent induced tumor growth inhibition, tumor regression only occurred when gedatolisib was combined with palbociclib, with or without fulvestrant^60^. In addition, results from a phase 1b clinical trial of gedatolisib in combination with the palbociclib and hormonal therapy demonstrated encouraging preliminary efficacy and tolerability independent of *PIK3CA* status^20^. These promising results led to the initiation of an ongoing Phase 3 clinical trial (VIKTORIA-1, NCT05501886) evaluating gedatolisib plus fulvestrant, with and without palbociclib, in patients previously treated with CDK4/6 and an aromatase inhibitor. The present study only compared multi-node and single-node PAM inhibitors as single agents in BC cell lines. In future non-clinical studies, it will be relevant to test if multi-node PAM inhibitors are more efficacious than single-node PAM inhibitors in combination with fulvestrant and palbociclib in BC cells.

Dysregulation of the PAM pathway in cancer cells leads to metabolic reprogramming and activation of multiple tumor-promoting functions. Differences between the functional and metabolic state of normal and cancer cells can be exploited by PAM inhibitors to affect metabolic functions predominantly utilized by tumor cells whether in early, less mutated cancer or later, more highly mutated cancer. This study highlights the importance of inhibiting multiple nodes of the PAM pathways to effectively control PAM functions critical for cancer cells survival and proliferation and achieve anti-proliferative and cytotoxic effects on different BC cell types.

## Methods

### CELsignia PI3K signaling pathway test on breast cancer primary cultures

Breast cancer primary cultures were established as previously described^61^ from tumor tissue samples obtained from de-identified, human breast tumors. Multiple clinical sites in the United States provided the tumor tissue samples. Tumor tissue acquisition was obtained upon written informed consent by each patient in compliance with all relevant ethical regulations including the Declaration of Helsinki and approved by the ethics committees of each participating site. IRB exemption was granted by Liberty IRB (Columbia, MD) after determining that the research did not involve human subjects as defined under 45 CFR 46.102(f). For the CELsignia test, low passage primary breast cancer cells were counted using a NucleoCounter NC-250 (Chemometec) and seeded into 96-well E-plates (Agilent) coated with collagen 1 (Advance Biomatrix) and fibronectin (Sigma). Real-time live cell responses to LPA receptor agonist 1-oleoyl lysophosphatidic acid (LPA) (Tocris) with and without antagonists (gedatolisib, alpelisib, or capivasertib [Selleckchem]) were measured and quantified using an xCELLigence RTCA impedance biosensor (Agilent) as described previously^61,62^. Following 18 h of antagonist treatment, cells were treated with 125 nM LPA and impedance changes were recorded for an additional 4 h. Impedance data analysis was performed using TraceDrawer (Ridgeview Instruments AB) to derive reported values in 4 h signaling units. LPA signal inhibition by the antagonists was calculated as previously described^61^.

### Cell line culture

The BC cell lines used in this study were obtained from commercial sources as listed in Supplementary Table 2. Cell lines were authenticated by STR profiling (ATCC) and tested for mycoplasma contamination. The KPL1 breast cancer cell line is a clonal derivative of the MCF7 breast cancer cell line with distinct genotypic and phenotypic features (CCLE analysis and Saunus et al.^63^). Cells were maintained in a humidified incubator at 37 °C and 5% CO_2_ based on the vendor’s recommendations. Cells were passaged when ~75–80% confluent and used for experiments within 2–3 passages from thawing. Cell lines’ driver alterations in PAM pathway genes were identified by cBioPortal (https://www.cbioportal.org/) analysis of the Cancer Cell Line Encyclopedia (CCLE, Broad 2019 dataset)^64^. The term ‘wild type (wt)’ is used here to define the absence driver alterations. Cell line tumor type and subtype, estrogen receptor (ER) status, and HER2 status are based on Dai et al. ^65^

### Treatments with PAM Inhibitors

Gedatolisib, alpelisib, capivasertib, everolimus, and copanlisib used for in vitro treatments were obtained from Selleckchem. Drugs were reconstituted at high concentration in DMSO and stored at −80 °C. Drugs were further diluted in DMSO and stored in aliquots at −30 °C for cell treatments. Cells were seeded in duplicate on white collagen 1/fibronectin-coated 96-well plates in 180 µl culture medium and let attach overnight. The seeding density of each cell line was optimized to ensure untreated cells remained in the growth phase throughout the assay. After attachment, cells were treated with PAM inhibitors at the indicated concentrations by adding 20 µl of 10× drug freshly diluted in medium. Control cells were treated with the same amount of DMSO used for drug treatments. Additional wells were seeded to obtain pre-treatment viability measurements for GR metrics calculations.

### Viability and cell death assay

Cells treated with or without PAM inhibitors for 72 h were analyzed for cell viability and cell death by using the RT-Glo MT luciferase assay (Promega) and Sytox Green (Thermo Fisher) staining, respectively. The 72-h treatment time was chosen based on previous studies^13^. A solution of RTGlo MT enzyme and substrate (both diluted 1:600) and Sytox Green (7.5 µM) was prepared in warm medium, and 40 µl/well were added to 96-well plates containing 200 µl medium/well. After 1–1.5-h incubation in a cell culture incubator at 37 °C and 5% CO_2_, an Infinite M1000 (Tecan) microplate reader was used to measure RTGlo MT luminescence (live cells) and Sytox Green fluorescence (dead cells) with excitation = 504 nm and emission = 523 nm. Cells were then lysed by addition of 10 µl/well of 10% Triton X 100 in PBS (Sigma), and fluorescence was measured again after incubation at 37 °C cell for 1–1.5 h to obtain a measurement of the total cells. Wells with culture medium + RTGlo MT/Sytox Green mix were used for background subtraction. Background-subtracted luminescence readings were normalized to DMSO-treated cells (set as 1) to obtain relative viability values. The background-subtracted Sytox Green readings were used to calculate the percent of dead cells using the formula FD/FT*100, where FD = fluorescence dead cells (before lysis) and FT = fluorescence total cells (after lysis). Pre- and post-lysis Sytox Green values were also used to calculate the number of live cells by subtracting the dead cell signal (before lysis) from the total cell signal (after lysis). Dose response curves (DRCs) and absolute IC_50_ values were calculated using PRISM (GraphPad Software). Cells with gedatolisib IC_50_ < 100 nM were considered sensitive to gedatolisib. Sensitivity cutoffs for alpelisib (3000 nM), capivasertib (3000 nM), and everolimus (50 nM) were based on previously published studies^35–37^.

### Proliferation-normalized inhibition of growth rate (GR) assays

RTGlo MT measurements before and after 72-h PAM inhibitor treatment were used to calculate normalized GR inhibition as described^23^. The normalized GR inhibition is calculated at time “t” in the presence of drug at concentration “c”, using the formula GR(c,t) = 2^k(c,t)/k(0)^ − 1 where k(c,t) is the growth rate of drug-treated cells and k(0) is the growth rate of untreated control cells. A GR value between 0 and 1 indicates an anti-proliferative effect; a GR value = 0 indicates complete cytostasis; a GR value between −1 and 0 indicates a cytotoxic effect. A dose response curve was used to assess drug potency and efficacy through the calculation of GR_50_ (concentration required to obtain a GR value = 0.5) and GR_Max_ (GR value at the maximal concentration tested), respectively. In addition, calculation of the area over the curve (GR_AOC_) was used to assess variations in potency and efficacy at the same time without the constraint of curve fitting^23^. PRISM was used to plot GR value DRCs and to calculate the GR_50_ of the various PAM inhibitors. GR_Max_ and GR_AOC_ were calculated with the online GR calculator tool^66^ using the same concentration range (1.4–9000 nM) for all PAM inhibitors.

### 3D culture assays

On the day of seeding, 96-well plates were coated with 50 µl of 60% Cultrex Reduced Growth Factor Basement Membrane Extract (BME), PathClear (R&D Systems) diluted in DMEM (Corning) and incubated in a humidified incubator at 37 °C and 5% CO_2_ for 30 min to induce BME polymerization. 10^4^ cells diluted in 130 µl culture medium + 2% BME were added to each well and incubated in a humidified incubator at 37 °C and 5% CO_2_ either overnight or for 3 days before drug treatment, as indicated. Additional wells were seeded in a separate plate to obtain measurements before drug treatment. Cells were treated in duplicate wells by adding 20 µl of 10× drug freshly diluted in culture medium. After 72 h of treatment, the medium was exchanged with culture medium + 2% BME + fresh drugs. After 6–7 days of treatment, cells were imaged by phase contrast microscopy and stained with 1.25 µM Sytox Green for 2 h at 37 °C. After Sytox green staining, fluorescence was measured with an Infinite M1000 (Tecan) microplate reader (Ex. = 504 nm Em. = 523 nm), and cells were imaged by fluorescence microscopy (Nikon E600). Cells were subsequently lysed by addition of 10 µl/well of 10% Triton X 100, and fluorescence was measured again after incubation at 37 °C cell for 2 h. The percentage of live and dead cells was calculated from the Sytox Green values as described above. The live cells Sytox Green values before and after treatment were used to calculate GR metrics as described above.

### Flow cytometry

After drug treatment for the indicated times, cells were harvested from 96-well plates for flow cytometry analyses. For the 5-ethynyl-2′-deoxyuridine (EdU) incorporation assay, cells were incubated with 10 µM EdU (Thermo Fisher) for the last 2 h of drug treatment. During this time, EdU (a nucleoside analog) is incorporated into newly synthesized DNA and can be used to assess DNA replication. For the OPP incorporation assay, cells were incubated with 5 µM OPP (Thermo Fisher) for the last 30 min of treatment. During this time, OPP (a puromycin analog) is incorporated into newly synthesized proteins and can be used to assess protein translation. At the end of the treatment, the medium (potentially containing floating dead cells) was collected and transferred to a deep-well 96-well plate. Cells were washed with PBS (Corning), incubated with 0.25%Trypsin (Corning) + 0.5 mM EDTA (Amresco) until cells detached, blocked with 0.3% Ovomucoid trypsin inhibitor (Worthington), and transferred to the deep-well 96-well plate along with the medium collected previously. After centrifugation at 300 g for 7 min at 4 °C, the cell pellets were washed with PBS, stained with Zombie NIR viability dye (Biolegend) for 15 min at room temperature, washed with PBS + 1%BSA, fixed with 1.6% paraformaldehyde for 10 min (Electron Microscopy Sciences), permeabilized with cold ACS grade methanol (Sigma) for 15 min, and used for different assays. For EdU incorporation and phospho-antibody staining, cells were stained by using the Click-iT EdU Alexa Fluor 647 kit (Thermo Fisher) according to the vendor’s instructions. After the Click-iT reaction, cells were washed with PBS + 1% BSA, stained with anti-pRPS6-BV421(S235/S236) (Biolegend) diluted 1:50 and anti-p4EBP1-Alexa Fluor 488 (T36/T45) (BD Biosciences) diluted 1:25 for 30 min at 4 °C, washed with PBS + 1% BSA, and run on a Novocyte 3005 (Agilent) flow cytometer. Data were analyzed by using NovoExpress 1.5.6 (Agilent). Cells were first gated by forward and side scatter to exclude cell debris. Live cells gated by Zombie staining were analyzed for EdU incorporation (% of EdU+ cells) and pRPS6 and p4EBP1 levels (median fluorescence intensity after unstained background subtraction). Data were normalized to DMSO-treated control cells (set at 1) and analyzed in PRISM to obtain DRCs and calculate IC_50_ values. Phospho-AKT (S473) was also used to assess PAM pathway activity; however, under our experimental conditions, pRPS6 and p4EBP1 were more robust and stable markers of PAM pathway activity and better correlates of cell viability outcomes than pAKT(S473) (data not shown). For EdU incorporation, apoptosis, and cell cycle analysis, cells were harvested, fixed, permeabilized, and stained with Click-iT EdU Alexa Fluor 647 kit as described above, incubated with anti-cleaved caspase 3-Alexa Fluor 488 (Cell Signaling) diluted 1:25 for 30 min at 4 °C, washed with PBS + 1% BSA, incubated with 1 µg/mL FxCycle Violet (Thermo Fisher) at 4 °C for at least 30 min, and analyzed by flow cytometry. Cell cycle phases were gated by plotting EdU incorporation (identifying DNA synthesis) versus DNA content (assessed by FxCycle). Cleaved Caspase 3-positive cells were analyzed in all cells (Zombie+ and Zombie-) using DMSO-treated cells to set the gate. For OPP incorporation and phospho-antibody staining, cells were stained by using the Click-iT OPP Alexa Fluor 647 kit (Thermo Fisher) based on the vendor’s instructions and previously published protocols^67^. After the Click-iT reaction, cells were stained with anti-pRPS6-BV421 and anti-p4EBP1-Alexa Fluor 488 as described above. Live cells gated by Zombie staining were analyzed for OPP incorporation and pRPS6 and p4EBP1 levels using the median fluorescence intensity after unstained background subtraction. Data were normalized to DMSO-treated control cells (set at 1) and analyzed in PRISM. Flow cytometry gating strategies are shown in Supplementary Figs. 11–13.

### Cell migration and invasion assays

Cell migration and invasion were assessed using transwell assays with permeable inserts for 24-multiwell plate with 8 µm pores (Corning). Uncoated inserts were used for the migration assay and Matrigel-coated BioCoat inserts were used for the invasion assay following the manufacturer’s recommendations. 5 × 10^4^ cells were resuspended in 0.5 mL FBS-free growth medium and added to the inserts (upper chamber of the transwell assay). Adjacent wells (lower chamber of the transwell assay) contained 0.75 mL growth media supplemented with 10% FBS as migration/invasion stimulus/attractant. Drugs at the indicated doses were added to both the upper and the lower chamber. Two hours after cells were seeded, the insert was moved to the adjacent wells containing the FBS-supplemented medium. The cells were then allowed to migrate from the upper chamber to the lower chamber for approximately 16 h (migration) or 24 h (invasion). After removing the cells from the upper chamber with a cotton swab, the bottom of the insert containing the migrating/invading cells was washed, fixed, and stained with a 1% crystal violet aqueous solution (Sigma-Aldrich). After imaging the migrating/invading cells by microscopy, the crystal violet stain was eluted with 33% acetic acid and quantified using an Infinite M1000 (Tecan) microplate reader at an absorbance of 590 nM.

### Oxygen consumption rate (OCR) analysis

OCR was measured using the Resipher instrument (Lucid Scientific, Inc.). Cells were seeded in a 96-well plate coated with collagen-fibronectin in a final media volume of 100 µL and allowed to attach for 24 h. 10 µL of an 11× drug freshly diluted in medium was added to the plate and the data collection proceeded on the Resipher for approximately 18 h. Control cells were treated with the same amount of DMSO used for drug treatments. Real time oxygen levels and OCR were analyzed with the LucidLab online application (Lucid Scientific). At the end of the 18-h period, the plate was removed from the instrument, and live cell number was quantified by flow cytometry using Sytox blue stain (Thermo Fischer) to exclude dead cells. The OCR at the end of the experiment was normalized to the number of live cells. The number of live cells did not change or changed only modestly after PAM inhibitor treatment.

### Glucose and lactate analysis

Glucose and lactate levels were measured using the Biosen R-line instrument (EKF Diagnostic Holdings). Cells were seeded in 96-well plates coated with collagen-fibronectin and allowed to attach for 48 h prior to drug addition. Growth media was removed and replaced with 100 µL fresh growth media, and then 10 µL of a solution of 11× drug freshly diluted in the medium was added to the cells. Control cells were treated with the same amount of DMSO used for drug treatments. After 24 h of treatment, a conditioned medium was collected for measuring glucose and lactate levels. 10 µL of collected media was added to 500 µL glucose/lactate hemolyzing solution (EKF Diagnostics), mixed by vortexing, and processed on the Biosen R-line instrument. Glucose consumption was assessed by subtracting the glucose level in the conditioned medium from baseline medium glucose level; lactate production was assessed by subtracting the baseline medium lactate level from the lactate level in the conditioned medium. The glucose consumption and lactate production values were normalized to cell number, which was assessed by RTglo MT assay (Promega) at the end of the treatment, as described above. The assessment of cell viability after treatment showed that the number of cells did not change or changed only modestly after PAM inhibitor treatment.

### Animal studies

Mini-PDX studies were performed by LideBiotech (Shanghai, China) based on Zhang et al.^28^. in compliance with ethical regulations for animal testing and in accordance with the regulations of the Association for Assessment and Accreditation of Laboratory Animal Care (AAALAC). The study protocol was approved by the Animal Welfare Committee of Shanghai LIDE. Female BALB/c nude mice (6–8 weeks old) were used for the study. Mice were kept in polycarbonate cages in a temperature and humidity-controlled environment with 12 h light and 12 h dark with free access to sterilized food and water. Tumor samples from the PDX mouse models were collected separately in cooled Hank’s balanced salt solution, cut into 1–3 mm^3^ pieces and digested with a collagenase solution at 37 °C for 1–2 h. Digested tissue was strained through a 70 µM strainer to obtain a single tumor cell suspension. Cells were centrifuged and re-suspended in RPMI1640 base medium (Gibco) and counted. The single-cell suspension was used to fill Mini-PDX capsules to implant into mice. The mice were randomized into groups based on body weight. The mini-PDX capsules were implanted into both flanks of BALB/c Nude mice previously anesthetized with isoflurane (1–2.5% inhalation) by making a 2–3 mm skin incision and using an 11–13 gauge trocar. The inoculation day was defined as day 0. Six capsules (3 capsules per mouse × 2 mice) were used for each arm of the study. Mice were treated with gedatolisib (human clinical formulation, Celcuity), alpelisib, capivasertib, everolimus (all from Selleckchem), or saline (vehicle control) for 7 days. Gedatolisib was resuspended in H_2_O and administered intravenously Q4D; alpelisib was resuspended in 5% DMSO (Sigma) + 40% PEG300 (Aladdin) + 5% Tween 80 (MeilunBio) + 50% ddH_2_O and administered PO QD; capivasertib was resuspended in 10% DMSO + 25% w/v Kleptose HPB (Roquette) buffer (Aladdin) and administered PO BID on a 4 days on, 3 days off schedule; everolimus was resuspended in 30% PEG300 + 5% Tween 80 + 65% ddH_2_O and administered PO QD. Mice were weighed every day and monitored for signs of morbidity. Mice did not show any sign of morbidity until the end of the 7-day treatment, which was the pre-established end-point of the experiment. At the end of the treatment, mice were euthanized by carbon dioxide inhalation in a euthanasia chamber, followed by cervical dislocation. Mini-PDX capsules were immediately removed, and the encapsulated tumor cell number was evaluated by CellTiter-Glo Luminescent 3D Cell Viability Assay (Promega) using an Infinite M Plex plate reader (TECAN). Tumor cell growth (%) was calculated from luminescence readings at day 0 and day 7 using the following formula: (mean RLU of the treatment group on day 7 − mean RLU on day 0)/(mean RLU of the vehicle group on day 7 − mean RLU on day 0). Tumor cell viability at the end of the treatment was the only metric analyzed in the mini-PDX studies. Since tumor cells are encapsulated and cannot expand further after 7 days, mini-PDX experiments could not be extended past 7 days to assess mice survivability or other long-term effects. For comparison between the two groups, a two-sided unpaired Student’s *t* test was performed, with *p* ≤ 0.05 considered statistically significant.

### Statistical analyses

Statistical significance was calculated using PRISM (GraphPad) or Excel, as indicated in the figure legends. The effects of each PAM inhibitor in different cell line subpopulations (e.g., the effect of compounds in cell lines with altered vs wild type *PIK3CA*) were compared by Brown-Forsythe and Welch one-way analysis of variance (ANOVA) using Dunnet T3 correction for multiple comparisons. The effects of different PAM inhibitors within a specific cell line subpopulation were compared by matched one-way ANOVA with Geisser–Greenhouse correction and Dunnett’s multiple comparison test. *P*-values < 0.05 were considered significant.

### Reporting summary

Further information on research design is available in the Nature Research Reporting Summary linked to this article.

### Supplementary information

Supporting information

Reporting Summary

Supplementary Data 1-12

## Data Availability

All data are available in the main text or the supplementary materials. The datasets analyzed during the current study are available from the corresponding author upon reasonable request.
