# Supplementary material for: Gedatolisib shows superior potency and efficacy versus single-node PI3K/AKT/mTOR inhibitors in breast cancer models
Source: NPJ Breast Cancer. 2024 Jun 5;10:40. doi: 10.1038/s41523-024-00648-0 (PMC11153628; doi:10.1038/s41523-024-00648-0)
Supplement: Supplementary file 1 — Supporting information [file 41523_2024_648_MOESM1_ESM.pdf]

# Supplementary Materials for

## Gedatolisib shows superior potency and efficacy versus single-node PI3K/AKT/mTOR inhibitors in breast cancer models

Stefano Rossetti, Aaron Broege, Adrish Sen, Salmaan Khan, Ian MacNeil, Jhomary Molden, Ross Kopher, Stephen Schulz, Lance Laing

Correspondence to: llaing@celcuity.com

**This file includes:**

Supplementary Figures 1-13
Supplementary Tables 1-3

**Other Supplementary Materials for this manuscript include the following:**

Supplementary Data 1-12 (Excel file)

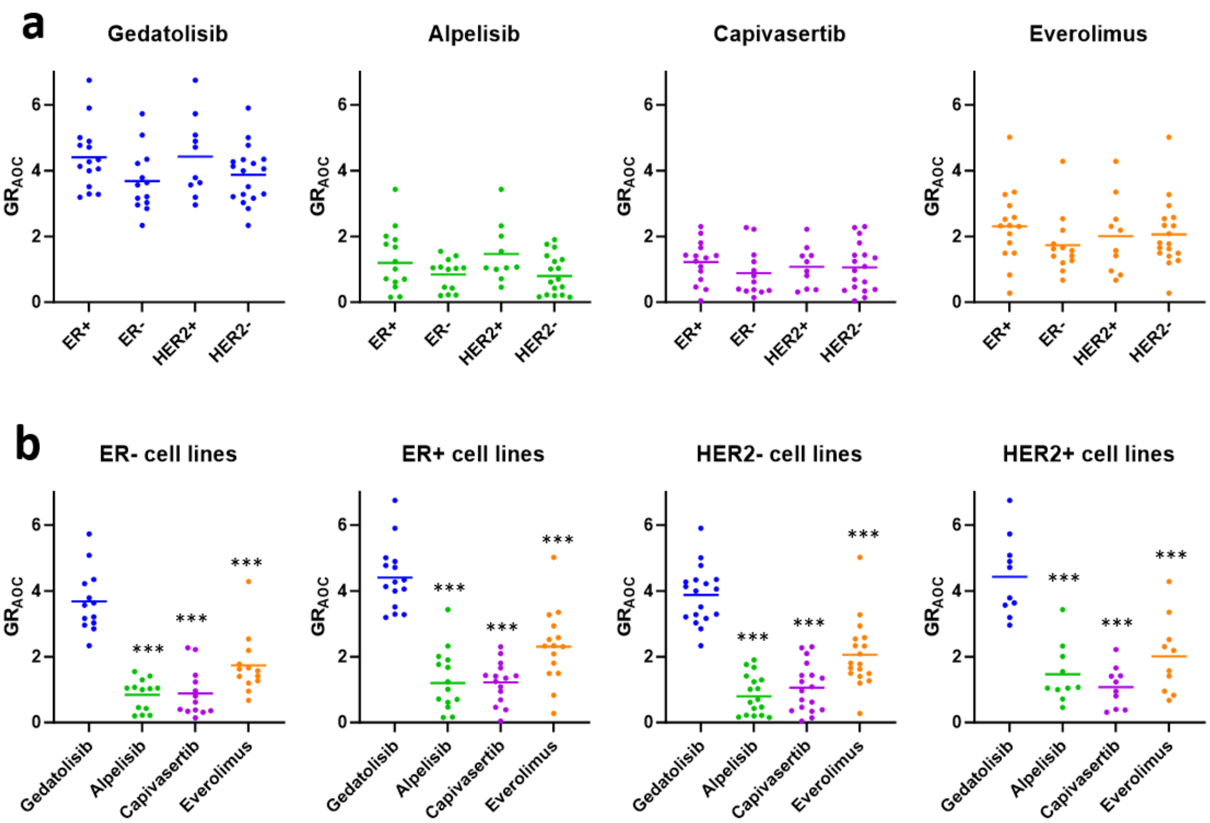


**Supplementary Figure 1. Comparison of PAM inhibitors $GR_{AOC}$ in BC cell lines with different ER and HER2 status. a.** The PAM inhibitors tested show similar potency and efficacy ($GR_{AOC}$) in ER+ vs ER- or HER2+ vs HER2- cell lines ($p > 0.05$ by Welch's t-test). **b.** Gedatolisib is more potent and efficacious than the other PAM inhibitors tested regardless of ER of HER2 status. *** = $p < 0.001$ vs gedatolisib by one-way ANOVA with Dunnet's multiple comparisons. GR = growth rate; AOC = area over the curve; ER = estrogen receptor

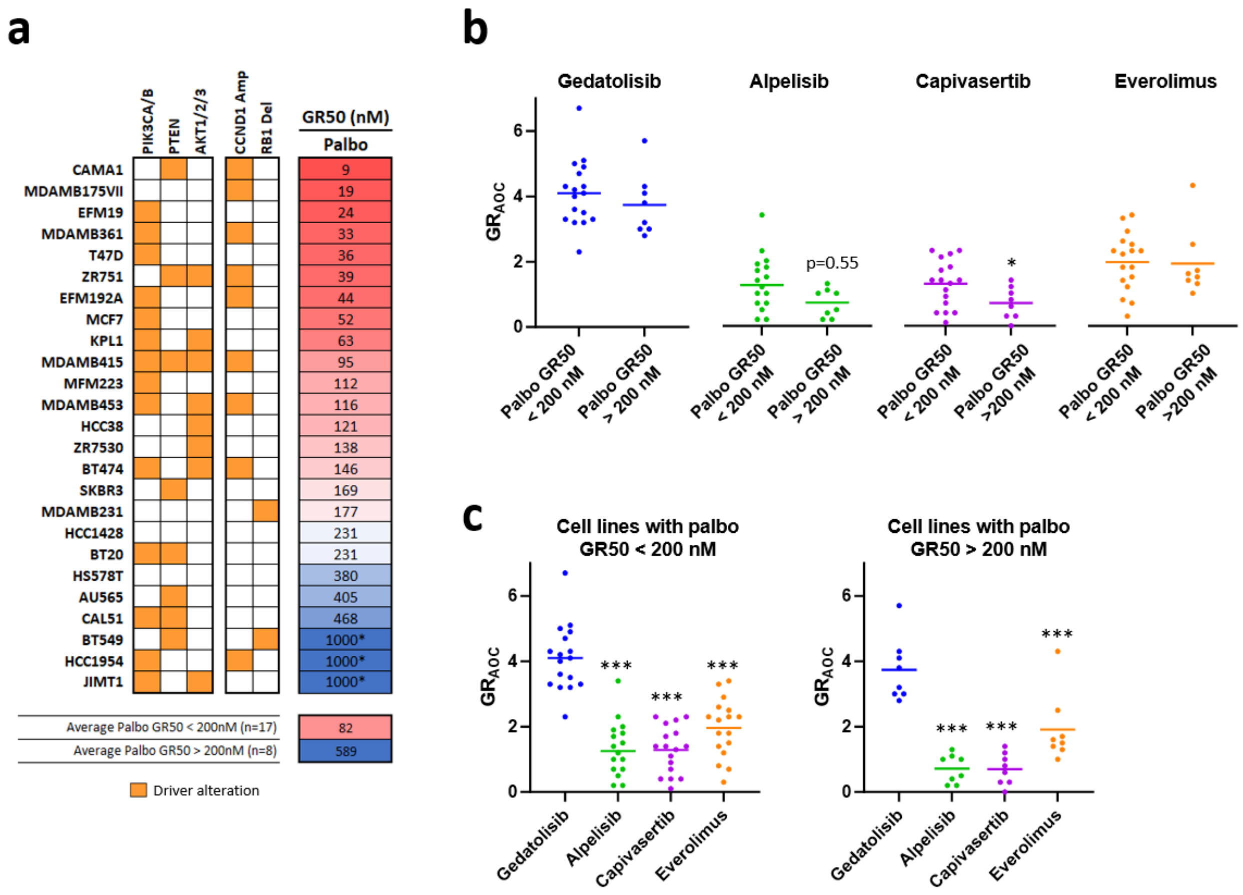


**Supplementary Figure 2. Comparison of PAM inhibitors $GR_{AOC}$ in BC cell lines with different sensitivity to palbociclib. a.** Palbociclib $GR_{50}$ in BC cell lines. Cells were treated with 1-1000 nM palbociclib for 6 days, exchanging the medium with fresh medium supplemented with fresh drug after 3 days. * = Max concentration tested, $GR_{50}$ not reached. **b.** Comparison of gedatolisib, alpelisib, capivasertib and everolimus $GR_{AOC}$ between cell lines with high or low sensitivity to palbociclib (palbociclib $GR_{50}$ < 200 nM = high sensitivity; palbociclib $GR_{50}$ > 200 nM = low sensitivity). * = $p < 0.05$ relative to palbo $GR_{50}$ <200 nM by Welch's t-test. **c.** $GR_{AOC}$ comparisons showing that gedatolisib is more potent and efficacious than the other PAM inhibitors tested regardless of palbociclib sensitivity. *** = $p < 0.001$ vs gedatolisib by one-way ANOVA with Dunnet's multiple comparisons. Del= deleted; Amp = amplified; GR = growth rate; AOC = area over the curve; palbo = palbociclib.

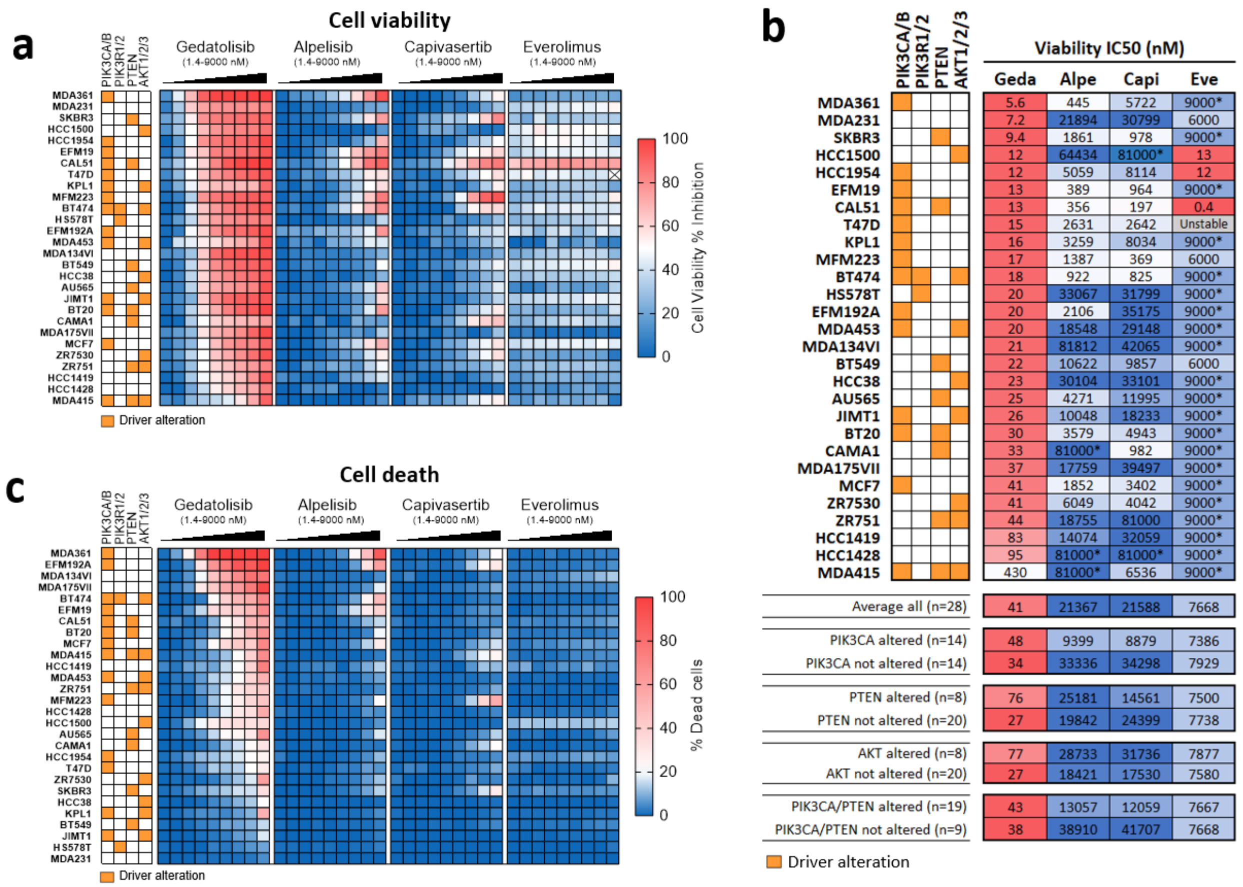


| | Viability IC50 (nM) | | | |
|---|---|---|---|---|
| | Geda | Alpe | Capi | Eve |
| MDA361 | 5.6 | 445 | 5722 | 9000* |
| MDA231 | 7.2 | 21894 | 30799 | 6000 |
| SKBR3 | 9.4 | 1861 | 978 | 9000* |
| HCC1500 | 12 | 64434 | 81000* | 13 |
| HCC1954 | 12 | 5059 | 8114 | 12 |
| EFM19 | 13 | 389 | 964 | 9000* |
| CAL51 | 13 | 356 | 197 | 0.4 |
| T47D | 15 | 2631 | 2642 | Unstable |
| KPL1 | 16 | 3259 | 8034 | 9000* |
| MFM223 | 17 | 1387 | 369 | 6000 |
| BT474 | 18 | 922 | 825 | 9000* |
| HS578T | 20 | 33067 | 31799 | 9000* |
| EFM192A | 20 | 2106 | 35175 | 9000* |
| MDA453 | 20 | 18548 | 29148 | 9000* |
| MDA134VI | 21 | 81812 | 42065 | 9000* |
| BT549 | 22 | 10622 | 9857 | 6000 |
| HCC38 | 23 | 30104 | 33101 | 9000* |
| AU565 | 25 | 4271 | 11995 | 9000* |
| JIMT1 | 26 | 10048 | 18233 | 9000* |
| BT20 | 30 | 3579 | 4943 | 9000* |
| CAMA1 | 33 | 81000* | 982 | 9000* |
| MDA175VII | 37 | 17759 | 39497 | 9000* |
| MCF7 | 41 | 1852 | 3402 | 9000* |
| ZR7530 | 41 | 6049 | 4042 | 9000* |
| ZR751 | 44 | 18755 | 81000 | 9000* |
| HCC1419 | 83 | 14074 | 32059 | 9000* |
| HCC1428 | 95 | 81000* | 81000* | 9000* |
| MDA415 | 430 | 81000* | 6536 | 9000* |
| Average all (n=28) | 41 | 21367 | 21588 | 7668 |
| PIK3CA altered (n=14) | 48 | 9399 | 8879 | 7386 |
| PIK3CA not altered (n=14) | 34 | 33336 | 34298 | 7929 |
| PTEN altered (n=8) | 76 | 25181 | 14561 | 7500 |
| PTEN not altered (n=20) | 27 | 19842 | 24399 | 7738 |
| AKT altered (n=8) | 77 | 28733 | 31736 | 7877 |
| AKT not altered (n=20) | 27 | 18421 | 17530 | 7580 |
| PIK3CA/PTEN altered (n=19) | 43 | 13057 | 12059 | 7667 |
| PIK3CA/PTEN not altered (n=9) | 38 | 38910 | 41707 | 7668 |

Driver alteration

**Supplementary Figure 3.** Analysis of BC cell viability and cell death in response to PAM inhibitors. **a.** Heatmap showing dose-dependent inhibition of cell viability (assessed by RTGlo MT assay) after 72-hour treatment with gedatolisib and other PAM inhibitors in 28 BC cell lines with various PAM pathway mutational status. % inhibition is relative to DMSO-treated cells. See Supplementary Data 2 for values. **b.** Cell viability absolute $IC_{50}$ values showing that gedatolisib is more potent than the other PAM inhibitors tested in most BC cell lines. Average $IC_{50}$ values in subpopulations with or without altered PAM pathway genes are shown. * = Max concentration tested, $IC_{50}$ not reached; Unstable = poor DRC fitting prevented reliable $IC_{50}$ calculation. **c.** Heatmap showing induction of cell death (assessed by SytoxGreen staining) after 72-hour treatment with gedatolisib and other PAM inhibitors in BC cell lines. See Supplementary Data 3 for values. geda = gedatolisib; alpe = alpelisib; capi = capivasertib; eve = everolimus

**a**

| Sample | Histology | ER/PR/HER2 status | Grade | Clinical stage | PTNM stage |
|---|---|---|---|---|---|
| C1076 | DCIS, Ductal Invasive | ER+, HER2 equivocal, PR- | 2 | II | pT3 pN0 |
| C1298 | DCIS, LCIS, Lobular Invasive | ER+, HER2-, PR+ | 2 | IIIA | pT3 pN1a |
| C1441 | DCIS, ductal invasive | ER+, HER2+, PR+ | 3 | IIA | pT2 pN0 |
| C1535 | DCIS | ER+, PR+ | 1 | 0 | pTis pN0 |

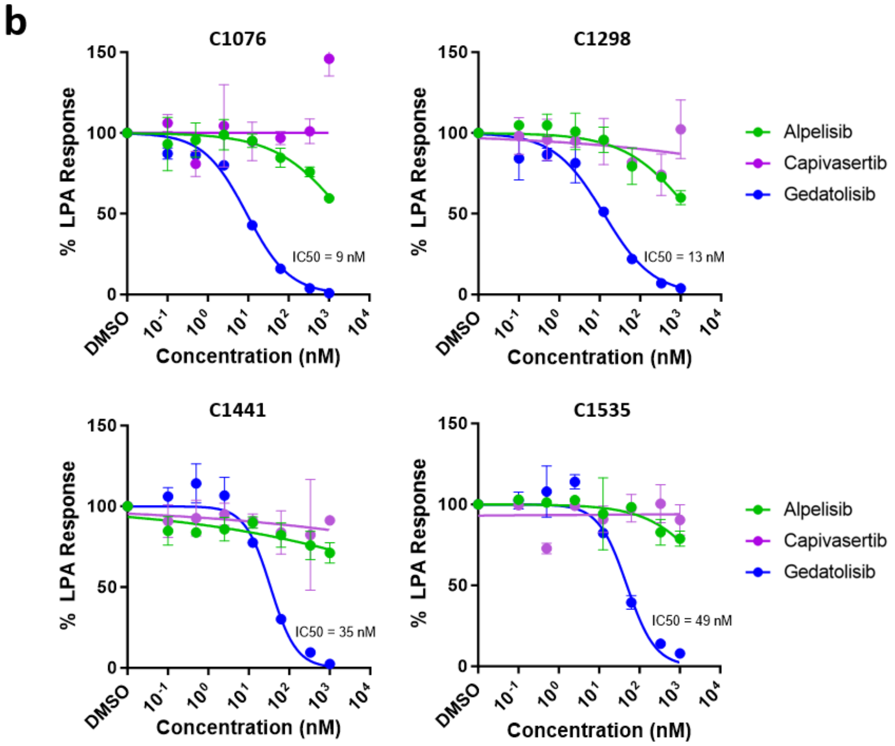


**Supplementary Figure 4.** CELsignia analysis of PAM inhibitors in BC primary cultures. **a.** Characteristics of the primary BC cultures tested with the CELsignia test. **b.** The CELSignia test shows that LPA-induced impedance is inhibited more effectively by gedatolisib than alpelisib and capivasertib in four BC primary cultures. Data represent mean +/- standard deviation (n=2 biologically independent samples). DCIS = ductal carcinoma in situ; LCIS = lobular carcinoma in situ; ER = estrogen receptor; PR = progesterone receptor; PTNM = pathological tumor-node-metastasis; LPA = lysophosphatidic acid.

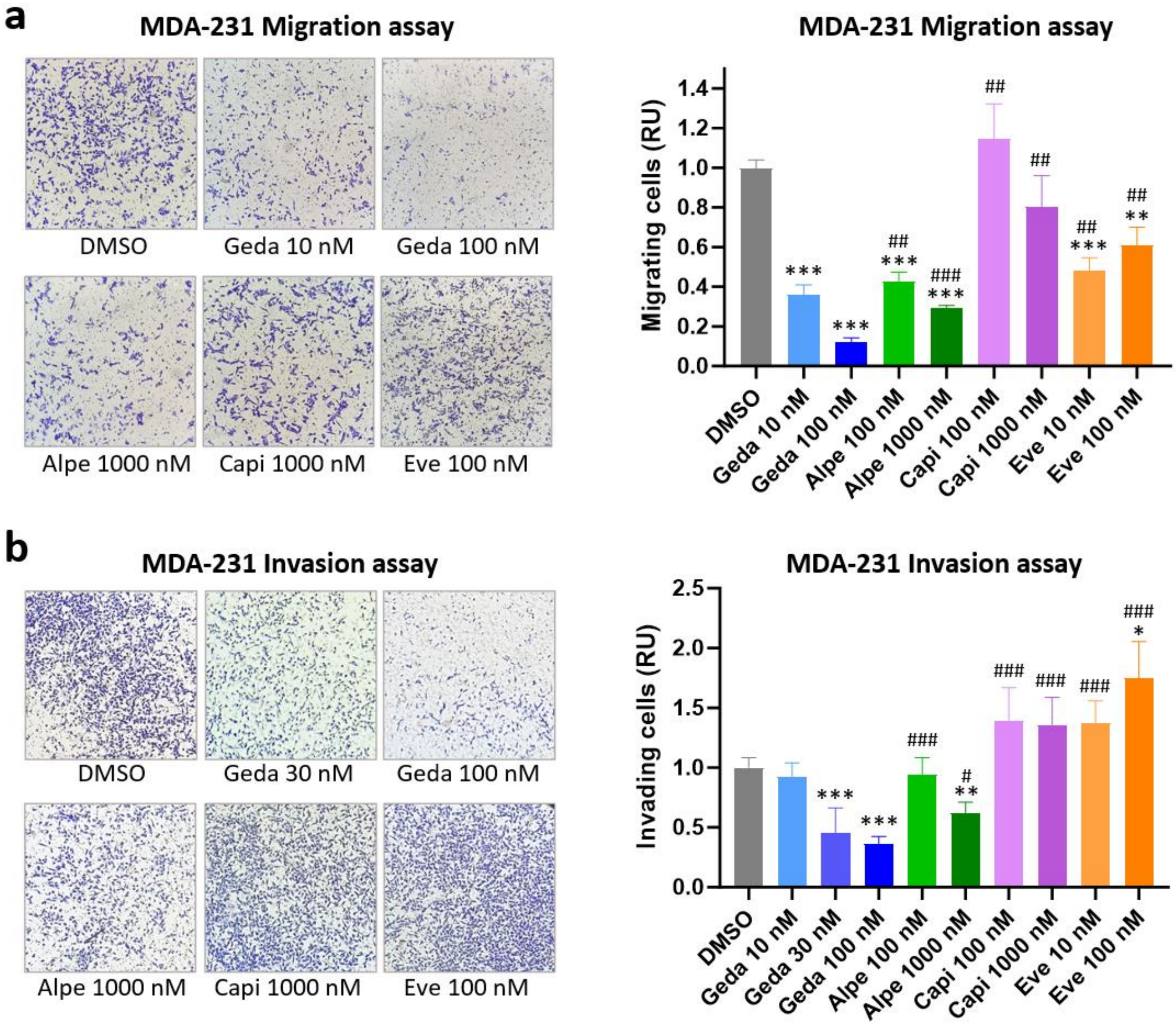


**Supplementary Figure 5.** Analysis of MDA-231 migration and invasion in response to PAM inhibitors. **a.** Transwell migration assay showing the effect of 16-hour treatment with PAM inhibitors at the indicated concentrations. Migrating cells were stained with crystal violet (left) and quantified by spectrometry after crystal violet elution (right). Data represent mean ± SEM (n = 3-4 biologically independent samples; see Supplementary Data 9 for individual values). **b.** Transwell invasion assay showing the effect of 24-hour treatment with PAM inhibitors at the indicated concentrations. Invading cells were stained with crystal violet (left) and quantified by spectrometry after crystal violet elution (right). Data represent mean ± SEM (n = 10-16 biologically independent samples; see Supplementary Data 10 for individual values). * p<0.05 **p<0.01 ***p<0.001 vs DSMO; $^{\#}$ p<0.05 $^{\#\#}$p<0.01 $^{\#\#\#}$ p<0.001 vs 100 nM gedatolisib by two-tailed, unpaired t-test. Geda = gedatolisib; alpe = alpelisib; capi = capivasertib; eve = everolimus; RU = relative units.

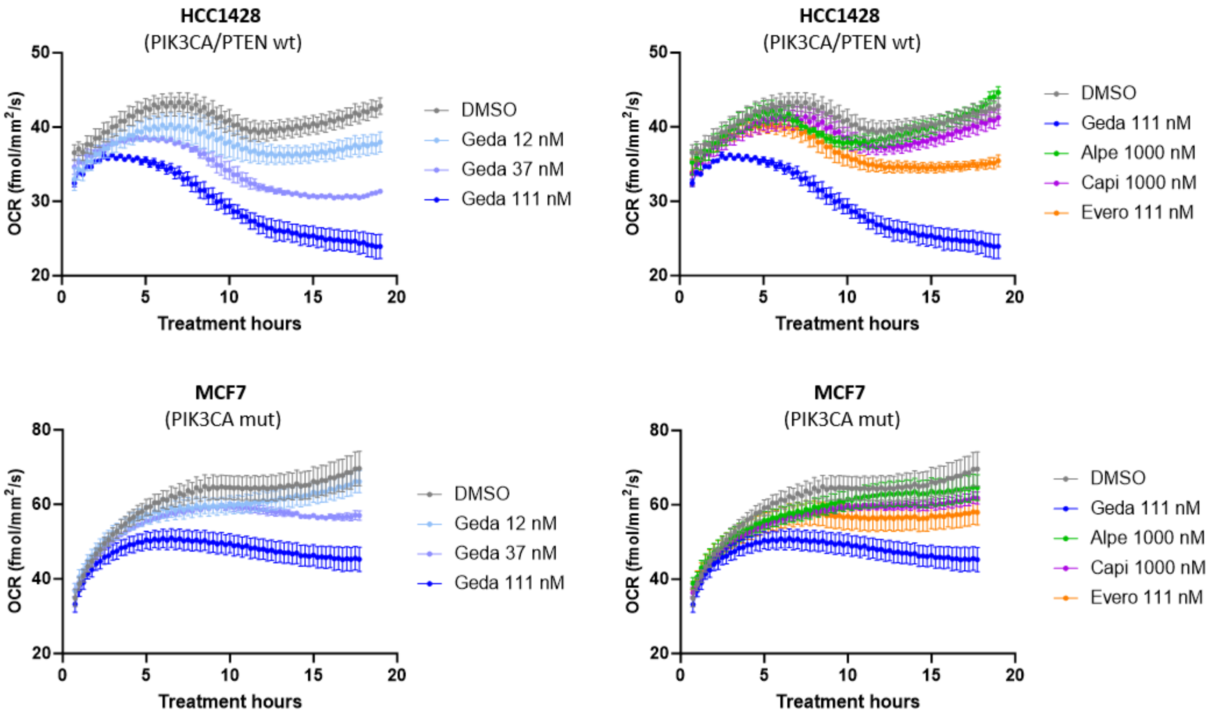


**Supplementary Figure 6.** Resipher analysis of OCR in HCC1428 and MCF7 cell lines treated with PAM inhibitors for approximately18 hours. OCR = oxygen consumption rate; wt = wild type; mut = mutant; geda = gedatolisib; alpe = alpelisib; capi = capivasertib; eve = everolimus.

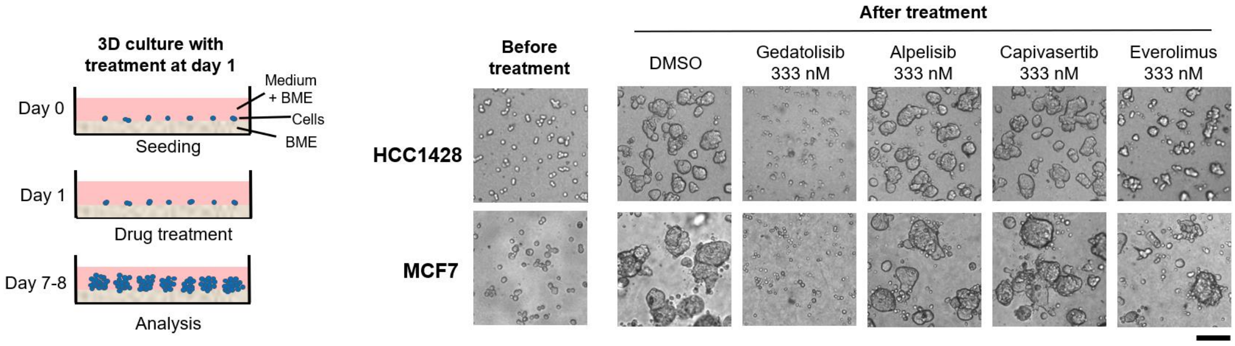


**Supplementary Figure 7. Analysis of PAM inhibitors in three-dimensional culture.** HCC1428 and MCF7 cells were grown in 3D culture on basement membrane extract (BME) for approximately 20 hours before treatment with PAM inhibitors for 6 days (HCC1428) or 7 days (MCF7) (left). Micrographs taken after treatment show that 333 nM gedatolisib inhibited 3D growth and induced spheroid regression more effectively than the other PAM inhibitors tested at the same concentration (right). Scale bar = 200 µm. 3D = three-dimensional; BME = basement membrane extract

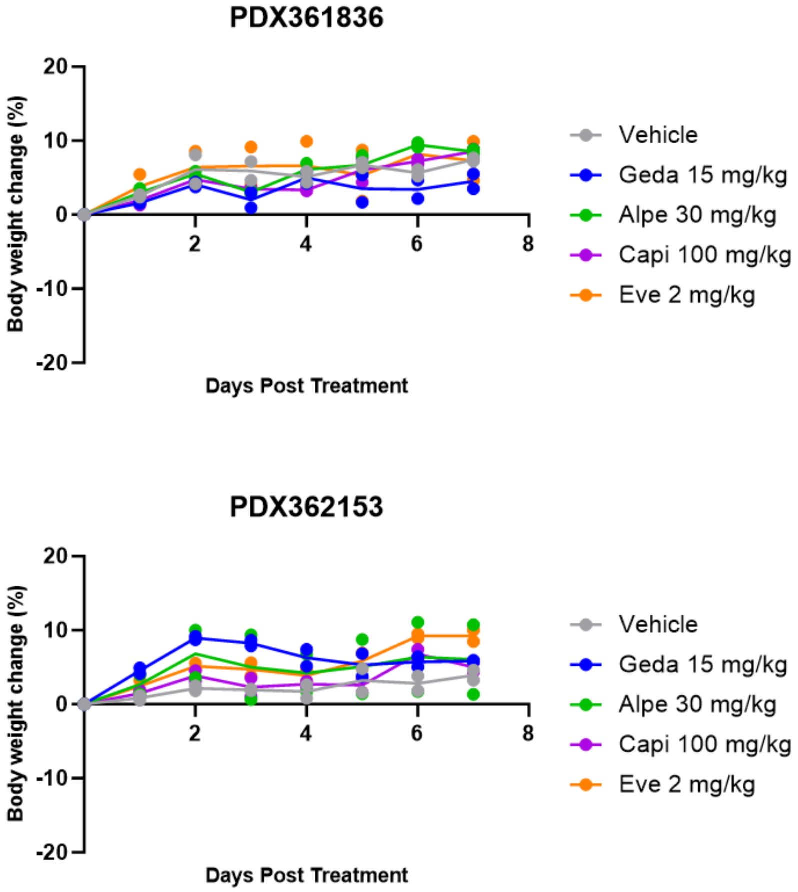


**Supplementary Figure 8** Analysis of PAM inhibitors in BC mini-PDX mouse models. Mice body weight measurements showed no significant change in body weight during the 7-day treatment with the PAM inhibitors. Gedatolisib: i.v. Q4D; alpelisib: p.o. QD; capivasertib: p.o. BID 4 days on/3 days off; everolimus: p.o. QD. Two mice per treatment (each implanted with 3 mini-PDX capsules).

**a**

| Drug | PAM specificity | Cell-free Assay Ki (nM) | | | | | |
|---|---|---|---|---|---|---|---|
| | | PI3Kα | PI3Kβ | PI3Kγ | PI3Kδ | mTOR | AKT1/2/3 |
| **Gedatolisib** | pan-PI3K, mTORC1/2 | 0.4 | 6 | 8 | 6 | 1 | - |
| **Alpelisib** | PI3Kα | 5 | >1000 | 250 | 290 | - | - |
| **Copanlisib** | pan-PI3K | 0.5 | 3.7 | 6.4 | 0.7 | 45 | - |

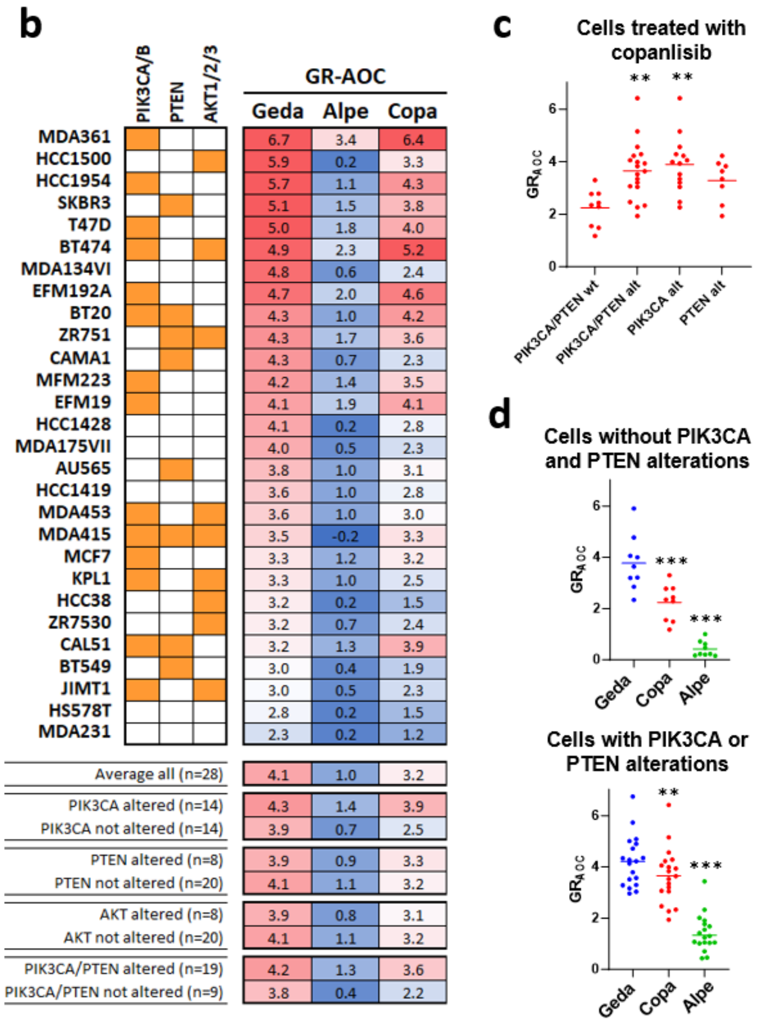


**Supplementary Figure 9. Comparison of gedatolisib, alpelisib and copanlisib $GR_{AOC}$ in BC cell lines. a.** Gedatolisib, alpelisib and copanlisib specificity. **b.** Analysis of $GR_{AOC}$ (area over the curve calculated from a 1.4-9000 nM drug titration) comparing potency and efficacy of gedatolisib (panPI3K/mTOR inhibitor), alpelisib (PI3Kα inhibitor), and copanlisib (panPI3K inhibitor) in 28 BC cells treated for 72 hours and analyzed by RTGlo MT luciferase assay. Higher $GR_{AOC}$ indicates higher potency and efficacy. **c.** Analysis of $GR_{AOC}$ showing that copanlisib is more potent and efficacious in cells lines with *PIK3CA* mutations. ** $p < 0.01$ vs *PIK3CA*/*PTEN* wild type by ANOVA Kruskal-Wallis test with Dunn's multiple comparison. **d.** Analysis of $GR_{AOC}$ showing that gedatolisib is, on average, more potent and efficacious than copanlisib in BC cell lines with or without *PIK3CA*/*PTEN* driver alterations ** $p < 0.01$, *** $p < 0.001$ vs gedatolisib by one-way ANOVA with Dunnet's multiple comparisons. geda = gedatolisib; alpe = alpelisib; copa = copanlisib; wt = wild type (i.e. no driver alterations); alt = altered; AOC = area over the curve.

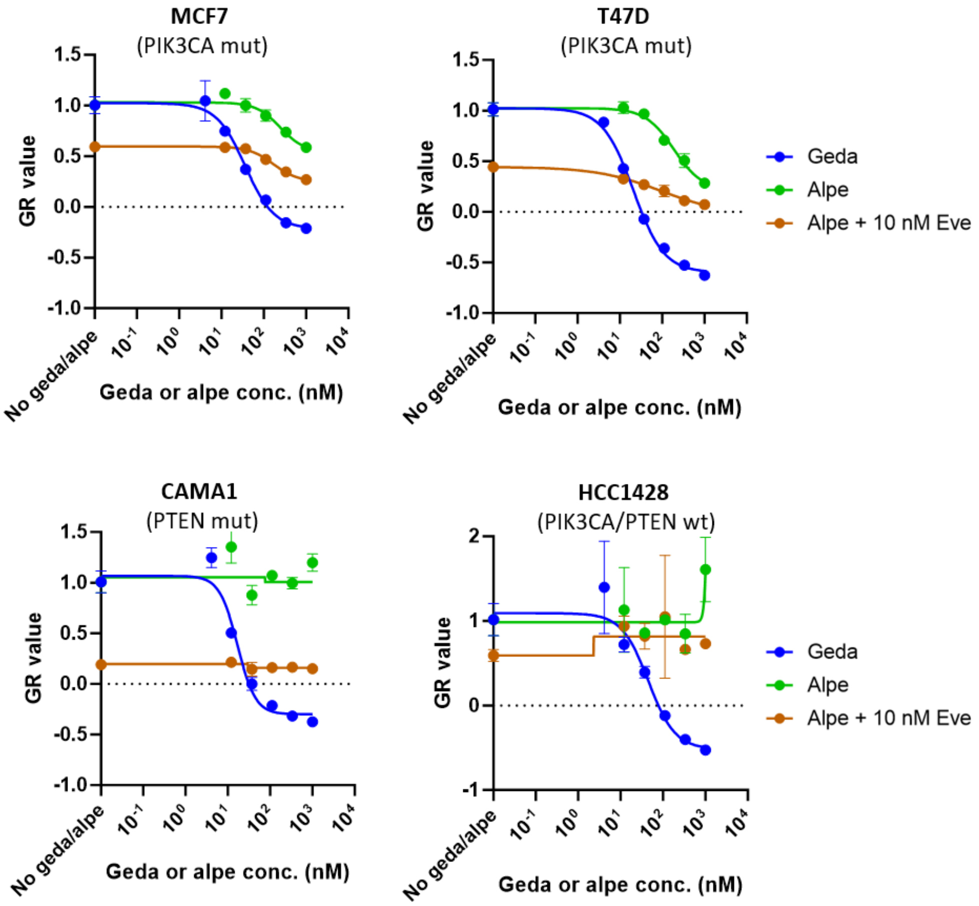


**Supplementary Figure 10. Concomitant inhibition of PI3Kα and mTORC1 by alpelisib and everolimus is less efficacious than panPI3K/mTORC1/mTORC2 inhibition by gedatolisib in BC cell lines.** Cells were treated with the indicated inhibitors for 72 hours and analyzed for GR metrics by RTGlo MT cell viability assay. Data represent mean +/- SD (n=2 biologically independent samples). Gedatolisib exerted cytotoxic effects (GR value <1) in all cell lines tested, while alpelisib exerted anti-proliferative effects (GR value between 0 and 1) only in *PIK3CA* mutant cell lines. Everolimus (10 nM) exerted an anti-proliferative effect (see GR value with no gedatolisib or alpelisib) in all cell lines. The addition of everolimus modestly increased the anti-proliferative effect of alpelisib in *PIK3CA* mutant lines but the combination of the two drugs did not reach the same level of efficacy observed with gedatolisib. GR= growth rate; geda = gedatolisib; alpe = alpelisib; eve = everolimus; wt = wild type; mut = mutant.

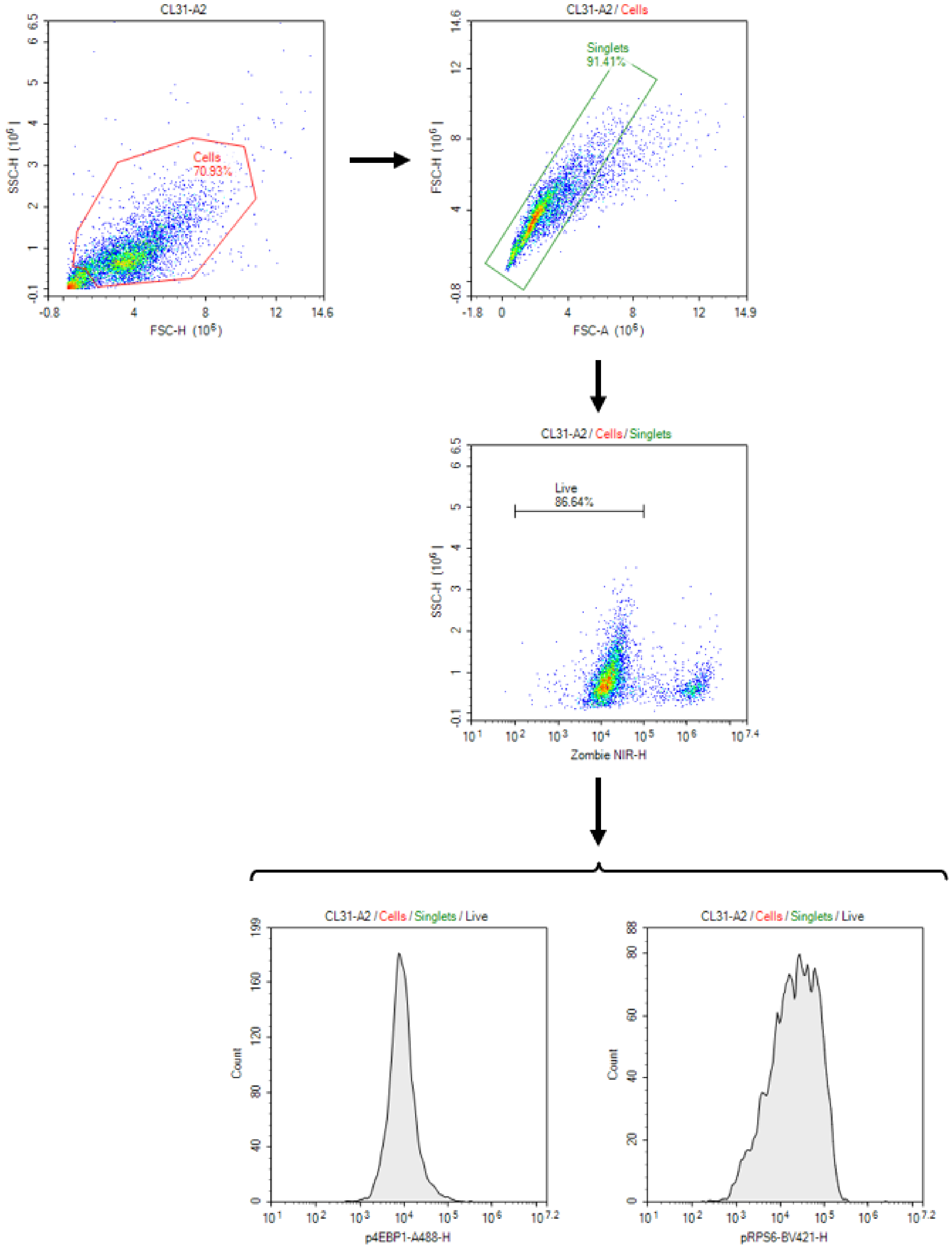


**Supplementary Figure 11.** Gating strategy used for flow cytometry analyses of Figure 2.

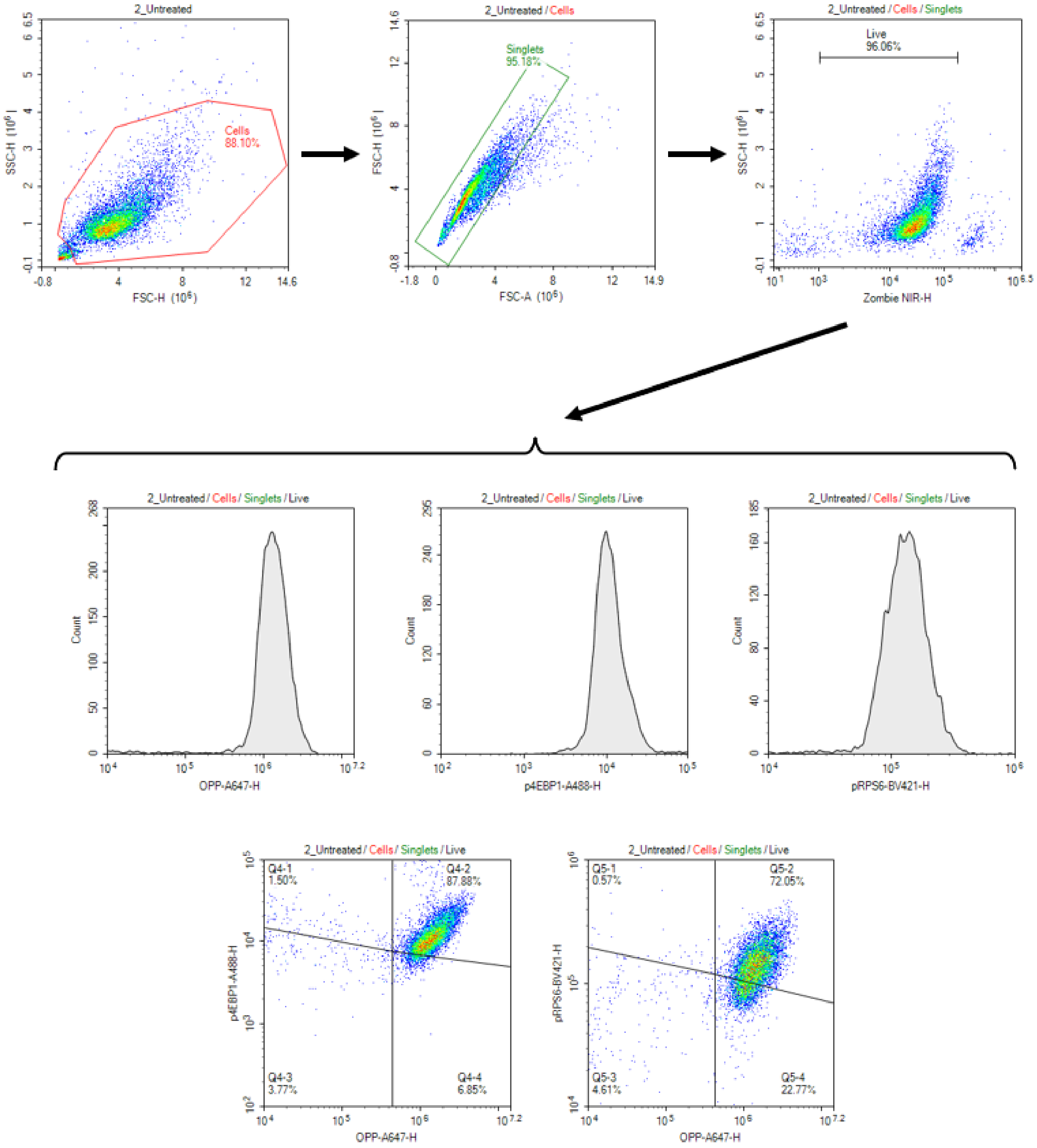


**Supplementary Figure 12.** Gating strategy used for flow cytometry analyses of Figure 3.

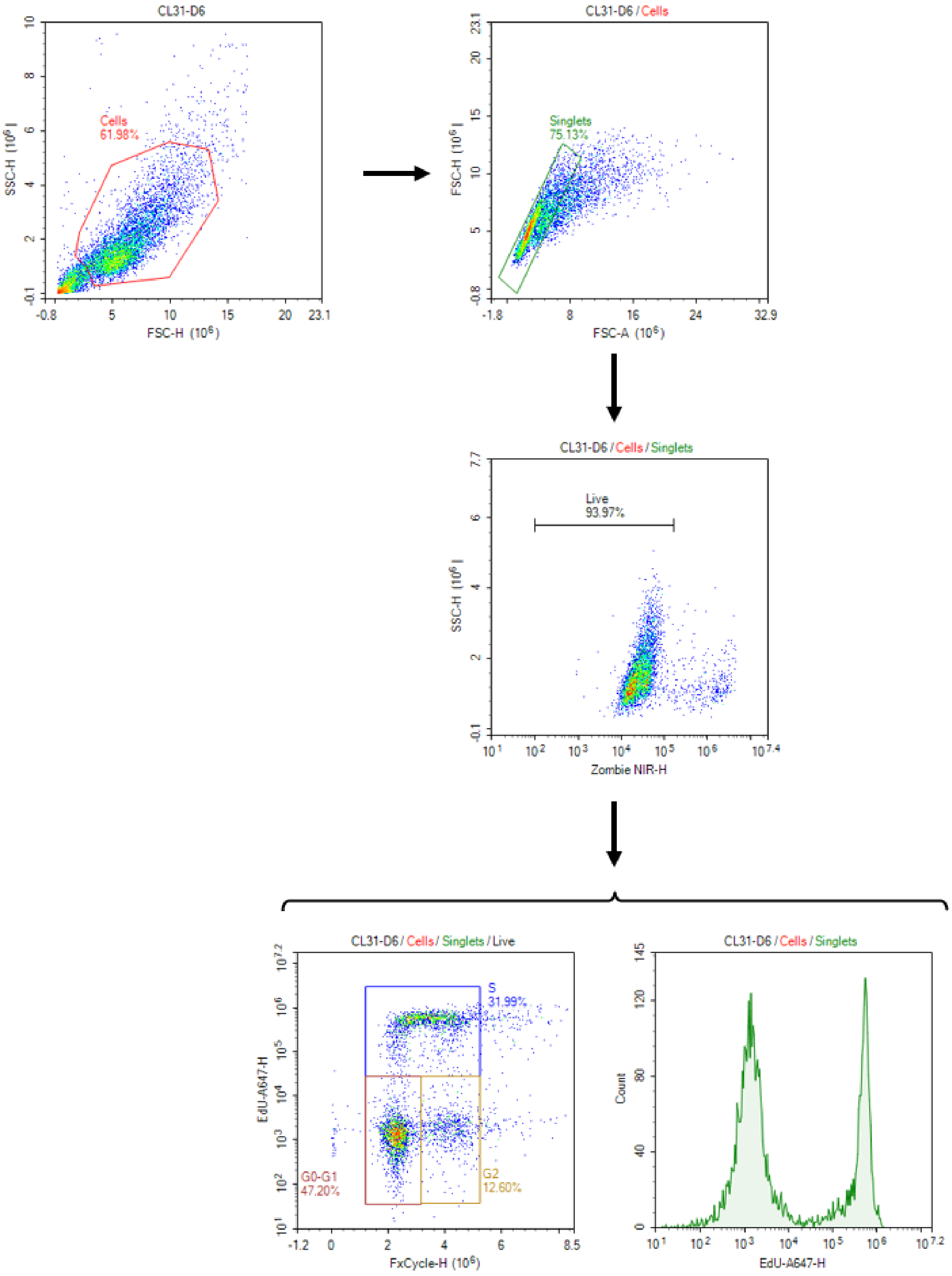


**Supplementary Figure 13.** Gating strategy used for flow cytometry analyses of Figure 4.

**Supplementary Table 1** Clinical trials evaluating gedatolisib as single agent or in combination with other anti-tumor agents

| NCT Number | Phase | Conditions | Interventions | N. patients | Study Status (04/2024) | Reference |
|---|---|---|---|---|---|---|
| **Single-Agent Gedatolisib** | | | | | | |
| NCT00940498 | Phase 1 | Neoplasms | Gedatolisib | 77 | Completed | Shapiro 2015; https://doi.org/10.1158/1078-0432.CCR-14-1306 |
| NCT01420081 | Phase 2 | Endometrial Neoplasms | Gedatolisib | 40 | Terminated | del Campo 2016; https://doi.org/10.1016/j.ygyno.2016.04.019 |
| NCT02142920 | Phase 1 | Healthy | Gedatolisib | 6 | Completed | Houk 2018; https://doi.org/10.1002/cpdd.615 |
| NCT02438761 | Phase 2 | AML and Myelodysplastic Syndrome | Gedatolisib | 10 | Terminated | Vargaftig 2018; http://doi.org/10.1182/blood-2018-99-117485 |
| **Gedatolisib in Combination with Other Anti-tumor Agents** | | | | | | |
| NCT01347866 | Phase 1 | Advanced Cancer | Gedatolisib+ irinotecan or PD-0325901 | 105 | Terminated | Wainberg 2017; https://doi.org/10.1007/s11523-017-0530-5 |
| NCT01920061 | Phase 1 | Advanced solid tumors and TNBC | Gedatolisib + docetaxel, cisplatin or dacomitinib | 107 | Completed | Curigliano 2023; https://doi.org/10.1038/s41416-022-02025-9 |
| NCT01925274 | Phase 2 | Metastatic Colorectal Cancer | gedatolisib + irinotecan | 19 | Terminated | Tabernero 2014; https://doi.org/10.1200/jco.2014.32.15_suppl.tps3649 |
| NCT01937715 | Phase 1/2 | Metastatic Colorectal Carcinoma | gedatolisib + FOLFIRI | 18 | Terminated | Wainberg 2014; https://doi.org/10.1200/jco.2014.32.15_suppl.tps3657 |
| NCT02069158 | Phase 1 | Advanced Solid Tumors | Gedatolisib+paclitaxel+carboplatin | 17 | Completed | Colombo 2021; https://doi.org/10.1158/1078-0432.CCR-21-1402 |
| NCT02626507 | Phase 1 | Breast Cancer | Gedatolisib+faslodex+palbociclib | 18 (estimated) | Unknown | NA |
| NCT02684032 | Phase 1 | Breast Cancer | Gedatolisib+palbociclib + fulvestrant or letrazole | 138 | Completed | Layman 2024; https://doi.org/10.1016/S1470-2045(24)00034-2 |
| NCT02920450 | Phase 1/2 | Non-Small Cell Lung Cancer | Gedatolisib + paclitaxel+carboplatin | 3 | Terminated | NA |
| NCT03065062 | Phase 1 | Advanced Solid Tumors | Gedatolisib+palbociclib | 96 (estimated) | Active | NA |
| NCT03243331 | Phase 1 | Metastatic TNBC | Gedatolisib+PTK7-ADC | 18 | Completed | Radovich 2022; https://doi.org/10.1158/1078-0432.CCR-21-3078 |
| NCT03698383 | Phase 2 | HER2-positive Metastatic Breast Cancer | Gedatolisib+herzuma | 15 | Unknown | Kim 2021; https://doi.org/10.1158/1538-7445.SABCS20-PS11-39 |
| NCT03911973 | Phase 1/2 | Advanced Breast Cancer | Gedatolisib+talazoparib | 37 | Active | Phadke 2022; https://doi.org/10.1200/JCO.2022.40.16_suppl.e13075 |
| NCT05501886 | Phase 3 | Advanced Breast Cancer | Gedatolisib+palbociclib+Fulvestrant | 701 (estimated) | Active | Hurvitz 2023; https://doi.org/10.1200/JCO.2023.41.16_suppl.TPS1118 |
| NCT06190899 | Phase 1/2 | Metastatic Castration-Resistant Prostate Cancer | Gedatolisib+Darolutamide | 54 (estimated) | Active | NA |

**Supplementary Table 2**. Cell lines used in this study

| Cell line | Source | Tumor type | Tumor subtype | ER+ | HER2+ | AKT1 | AKT2 | AKT3 | PIK3CA | PIK3CB | PIK3R1 | PIK3R2 | PTEN | TP53 |
|---|---|---|---|---|---|---|---|---|---|---|---|---|---|---|
| AU565 | ATCC | AC | H | - | Yes | - | - | - | - | - | - | - | HOMDEL | Mut |
| BT20 | ATCC | IDC | TNA | - | - | - | - | - | Mut | - | - | - | HOMDEL | Mut |
| BT474 | ATCC | IDC | LB | Yes | Yes | - | - | AMP | Mut | - | - | HOMDEL | - | Mut |
| BT549 | ATCC | IDC | TNB | - | - | - | - | - | - | - | - | - | Mut | Mut |
| CAL51 | DSMZ | AC | TNB | - | - | - | - | - | Mut | - | - | v | Mut | - |
| CAMA1 | ATCC | AC | LA | Yes | - | - | - | - | - | - | - | - | Mut | Mut, HOMDEL |
| EFM19 | DSMZ | IDC | LA | Yes | - | - | - | - | Mut | - | - | - | - | Mut |
| EFM192A | DSMZ | AC | LB | Yes | Yes | - | - | - | Mut | - | - | - | - | Mut |
| HCC1419 (1) | ATCC | IDC | NA | - | Yes | - | - | - | - | - | - | - | - | Mut |
| HCC1428 | ATCC | AC | LA | Yes | - | - | - | - | - | - | - | - | - | HOMDEL (dr) |
| HCC1500 (1) | ATCC | IDC | - | Yes | - | - | - | AMP | - | - | - | - | - | HOMDEL (dr) |
| HCC1954 | ATCC | DC | H | - | Yes | - | - | - | Mut | - | - | - | - | Mut |
| HCC38 | ATCC | DC | TNB | - | - | - | - | AMP | - | - | - | - | - | Mut, HOMDEL |
| HS578T | ATCC | IDC | TNB | - | - | - | - | - | - | - | Mut | - | - | Mut |
| JIMT1 | DSMZ | IDC | NA | - | Yes | - | - | - | Mut , AMP | AMP | - | - | - | Mut |
| KPL1 (2) | DSMZ | IDC | LA | Yes | - | AMP | - | - | Mut | - | - | - | - | - |
| MCF7 | ATCC | IDC | LA | Yes | - | - | - | - | Mut | - | - | - | - | - |
| MDAMB134VI | ATCC | IDC | LA | Yes | - | - | - | - | - | - | - | - | - | HOMDEL |
| MDAMB175VII | ATCC | IDC | LA | Yes | - | - | - | - | - | - | - | - | - | HOMDEL |
| MDAMB231 | ATCC | AC | TNB | - | - | - | - | - | - | - | - | - | - | Mut |
| MDAMB361 | ATCC | AC | LB | Yes | Yes | - | - | - | Mut | - | - | - | - | Mut |
| MDAMB415 | ATCC | AC | LA | Yes | - | - | - | AMP | AMP | - | - | - | Mut | Mut |
| MDAMB453 | ATCC | AC | H | - | Yes | - | - | AMP | Mut | - | - | - | - | HOMDEL |
| MFM223 | Sigma | C | TNA | - | - | - | - | - | Mut | - | - | - | - | Mut |
| SKBR3 | ATCC | AC | H | - | Yes | - | - | - | - | - | - | - | HOMDEL | Mut |
| T47D | ATCC | IDC | LA | Yes | - | - | - | - | Mut , AMP | - | - | - | - | Mut |
| ZR751 | ATCC | IDC | LA | Yes | - | - | - | AMP | - | - | - | - | Mut | HOMDEL |
| ZR7530 | ATCC | IDC | LB | Yes | Yes | - | - | AMP | - | - | - | - | - | HOMDEL |

AC=adenocarcinoma; C=carcinoma; DC=ductal carcinoma; IDC=invasive ductal carcinoma; LA = luminal A; LB=Luminal B; H=HER2+; TNA=Triple neg A; TNB=Triple Neg B; AMP = amplified; Mut = mutated; HOMDEL = deep deletion; Red = driver alteration

(1) ER status and tumor subtype inconsistently reported in the literature (Dai et al. 2017; PMID: 29158785)

(2) clonal derivative of MCF7 (Saunus 2018; PMID: 28889351)

**Supplementary Table 3**. PAM inhibitors potency and efficacy in breast cancer cell lines

## A. Cell line potency and efficacy based on GR metrics[1]

| | Average GR50 (nM) | | | | % Sensitive cell lines[2] | | | | Average GR-Max[3] | | | | % High efficacy cell lines[4] | | | |
|---|---|---|---|---|---|---|---|---|---|---|---|---|---|---|---|---|
| | Geda | Alpe | Capi | Evero[5] | Geda | Alpe | Capi | Evero | Geda | Alpe | Capi | Evero | Geda | Alpe | Capi | Evero |
| All cell lines (n=28) | 12 | 6308 | 8666 | 3611 | 100% | 57% | 54% | 46% | -0.68 | 0.11 | 0.20 | 0.38 | 96% | 43% | 29% | 7% |
| PIK3CA altered (n=14) | 12 | 2594 | 2590 | 1867 | 100% | 86% | 79% | 64% | -0.74 | -0.16 | 0.01 | 0.32 | 100% | 64% | 43% | 7% |
| PIK3CA wt (n=14) | 12 | 10308 | 15209 | 5501 | 100% | 29% | 29% | 29% | -0.62 | 0.38 | 0.40 | 0.44 | 93% | 21% | 14% | 7% |
| PTEN altered (n=8) | 14 | 4242 | 1894 | 2573 | 100% | 63% | 75% | 50% | -0.71 | 0.09 | -0.01 | 0.34 | 100% | 50% | 38% | 0% |
| PTEN wt (n=20) | 11 | 7178 | 11517 | 4015 | 100% | 55% | 45% | 45% | -0.67 | 0.12 | 0.29 | 0.40 | 95% | 40% | 25% | 10% |
| PIK3CA alt/PTEN alt (n=19) | 12 | 2783 | 2602 | 2134 | 100% | 79% | 74% | 58% | -0.72 | -0.10 | 0.00 | 0.33 | 100% | 63% | 42% | 5% |
| PIK3CA wt/PTEN wt (n=9) | 13 | 14681 | 23067 | 6752 | 100% | 11% | 11% | 22% | -0.59 | 0.55 | 0.63 | 0.49 | 89% | 0% | 0% | 11% |

## B. Cell line potency and efficacy based on cell viability[6]

| | Average IC50 (nM) | | | | % Sensitive cell lines[7] | | | | Max cell viability inhibition[8] | | | | % High efficacy cell lines[9] | | | |
|---|---|---|---|---|---|---|---|---|---|---|---|---|---|---|---|---|
| | Geda | Alpe | Capi | Evero[5] | Geda | Alpe | Capi | Evero | Geda | Alpe | Capi | Evero | Geda | Alpe | Capi | Evero |
| All cell lines (n=28) | 41 | 21367 | 21588 | 7668 | 96% | 32% | 25% | 11% | 92% | 53% | 47% | 31% | 100% | 54% | 46% | 14% |
| PIK3CA altered (n=14) | 48 | 9399 | 8879 | 7386 | 93% | 57% | 36% | 14% | 94% | 68% | 56% | 31% | 100% | 86% | 71% | 14% |
| PIK3CA wt (n=14) | 34 | 33336 | 34298 | 7929 | 100% | 7% | 14% | 7% | 89% | 37% | 37% | 31% | 100% | 21% | 21% | 14% |
| PTEN altered (n=8) | 76 | 25181 | 14561 | 7500 | 88% | 25% | 38% | 13% | 90% | 54% | 58% | 37% | 100% | 50% | 63% | 25% |
| PTEN wt (n=20) | 27 | 19842 | 24399 | 7738 | 100% | 35% | 20% | 10% | 92% | 52% | 42% | 29% | 100% | 55% | 40% | 10% |
| PIK3CA alt/PTEN alt (n=19) | 43 | 13057 | 12059 | 7667 | 95% | 47% | 37% | 11% | 92% | 64% | 56% | 32% | 100% | 74% | 63% | 16% |
| PIK3CA wt/PTEN wt (n=9) | 38 | 38910 | 41707 | 7668 | 100% | 0% | 0% | 11% | 90% | 29% | 27% | 29% | 100% | 11% | 11% | 11% |

[1]GR calculated from RTGlo MT assay before and after 72 hour treatment; [2]Sensitive cell lines: GR50 < 100 nM for gedatolisib; GR50 < 3000 nM for alpelisib, capivasertib; GR50 < 50 nM for everolimus; [3]Based on 1.4-9000 nM titration; [4]High efficacy cell lines: GRMax < 0 (max conc tested = 9000 nM); [5]Bimodal distribution; [6]Viability from RTGlo MT assay after 72 hour treatment (endpoint analysis); [7]Sensitive cell lines: IC50 <100 nM for gedatolisib; IC50<3000 nM for alpelisib, capivasertib; IC50 < 50 nM for everolimus; [8]Based on 1.4-9000 nM titration; [9]High efficacy cell lines: Emax > 50% viability inhibition (max conc tested = 9000 nM).
