# Supplementary material for: Gedatolisib shows superior potency and efficacy versus single-node PI3K/AKT/mTOR inhibitors in breast cancer models
Source: NPJ Breast Cancer. 2024 Jun 5;10:40. doi: 10.1038/s41523-024-00648-0 (PMC11153628; doi:10.1038/s41523-024-00648-0)
Supplement: Supplementary file 2 — Reporting Summary [file 41523_2024_648_MOESM2_ESM.pdf]

nature portfolio

Corresponding author(s): NPJBCANCER-03212

Last updated by author(s): May 8, 2024

Reporting Summary

Nature Portfolio wishes to improve the reproducibility of the work that we publish. This form provides structure for consistency and transparency in reporting. For further information on Nature Portfolio policies, see our Editorial Policies and the Editorial Policy Checklist.

Statistics

For all statistical analyses, confirm that the following items are present in the figure legend, table legend, main text, or Methods section.

n/a	Confirmed	
☐	☒	The exact sample size (*n*) for each experimental group/condition, given as a discrete number and unit of measurement
☐	☒	A statement on whether measurements were taken from distinct samples or whether the same sample was measured repeatedly
☐	☒	The statistical test(s) used AND whether they are one- or two-sided<br>*Only common tests should be described solely by name; describe more complex techniques in the Methods section.*
☐	☒	A description of all covariates tested
☐	☒	A description of any assumptions or corrections, such as tests of normality and adjustment for multiple comparisons
☐	☒	A full description of the statistical parameters including central tendency (e.g. means) or other basic estimates (e.g. regression coefficient) AND variation (e.g. standard deviation) or associated estimates of uncertainty (e.g. confidence intervals)
☒	☐	For null hypothesis testing, the test statistic (e.g. *F*, *t*, *r*) with confidence intervals, effect sizes, degrees of freedom and *P* value noted<br>*Give P values as exact values whenever suitable.*
☒	☐	For Bayesian analysis, information on the choice of priors and Markov chain Monte Carlo settings
☒	☐	For hierarchical and complex designs, identification of the appropriate level for tests and full reporting of outcomes
☒	☐	Estimates of effect sizes (e.g. Cohen's *d*, Pearson's *r*), indicating how they were calculated

Our web collection on statistics for biologists contains articles on many of the points above.

Software and code

Policy information about availability of computer code

Data collection	NovoExpress 1.5.6 (Agilent), LucidLab (Lucid Scientific); i-control 2.0 (TECAN)
Data analysis	Excel (Microsoft 365 for business); Prism 10 (GraphPad); NovoExpress 1.5.6 (Agilent); LucidLab (Lucid Scientific)

For manuscripts utilizing custom algorithms or software that are central to the research but not yet described in published literature, software must be made available to editors and reviewers. We strongly encourage code deposition in a community repository (e.g. GitHub). See the Nature Portfolio guidelines for submitting code & software for further information.

Data

Policy information about availability of data

All manuscripts must include a data availability statement. This statement should provide the following information, where applicable:

- Accession codes, unique identifiers, or web links for publicly available datasets
- A description of any restrictions on data availability
- For clinical datasets or third party data, please ensure that the statement adheres to our policy

All data are available in the main text or the supplementary materials. The dataset analyzed during the current study are available from the corresponding author upon reasonable request.

Research involving human participants, their data, or biological material

Policy information about studies with human participants or human data. See also policy information about sex, gender (identity/presentation), and sexual orientation and race, ethnicity and racism.

Reporting on sex and gender	Liberty IRB (Columbia, MD) determined that this study did not involve human subjects as defined under 45 CFR 46.102(f) and granted IRB exemption.
Reporting on race, ethnicity, or other socially relevant groupings	Liberty IRB (Columbia, MD) determined that this study did not involve human subjects as defined under 45 CFR 46.102(f) and granted IRB exemption.
Population characteristics	Liberty IRB (Columbia, MD) determined that this study did not involve human subjects as defined under 45 CFR 46.102(f) and granted IRB exemption.
Recruitment	Liberty IRB (Columbia, MD) determined that this study did not involve human subjects as defined under 45 CFR 46.102(f) and granted IRB exemption.
Ethics oversight	Liberty IRB (Columbia, MD) determined that this study did not involve human subjects as defined under 45 CFR 46.102(f) and granted IRB exemption.

Note that full information on the approval of the study protocol must also be provided in the manuscript.

Field-specific reporting

Please select the one below that is the best fit for your research. If you are not sure, read the appropriate sections before making your selection.

☒ Life sciences ☐ Behavioural & social sciences ☐ Ecological, evolutionary & environmental sciences

For a reference copy of the document with all sections, see nature.com/documents/nr-reporting-summary-flat.pdf

Life sciences study design

All studies must disclose on these points even when the disclosure is negative.

Sample size	Sample size was based on variance expected for specific metrics and on previous experiments showing that the sample size chosen was sufficient to obtain statistically significant differences
Data exclusions	Data were excluded in very few instances (indicated in the manuscript) due to technical issues or human error
Replication	Experiments were run with multiple independent biological replicates as indicated. Findings were also replicated in multiple cell lines. Replicate samples and replicate experiments showed similar trends.
Randomization	For animal studies, mice were randomized into groups based on body weight
Blinding	Blinding was not relevant to this study as all metrics and measurements were not influenced by the operator.

Reporting for specific materials, systems and methods

We require information from authors about some types of materials, experimental systems and methods used in many studies. Here, indicate whether each material, system or method listed is relevant to your study. If you are not sure if a list item applies to your research, read the appropriate section before selecting a response.

Materials & experimental systems

n/a	Involved in the study	
☐	☒	Antibodies
☐	☒	Eukaryotic cell lines
☒	☐	Palaeontology and archaeology
☐	☒	Animals and other organisms
☒	☐	Clinical data
☒	☐	Dual use research of concern
☒	☐	Plants

Methods

n/a	Involved in the study	
☒	☐	ChIP-seq
☐	☒	Flow cytometry
☒	☐	MRI-based neuroimaging

Antibodies

Antibodies used	Anti-pRPS6-BV421(S235/S236) (Biolegend, Cat # 608610, lot # B369141) ; anti-p4EBP1-AF488 (T36/T45) (BD Biosciences Cat# 560287, lot # 2139871); anti-cleaved caspase 3-A488 (Cell Signaling, Cat # 9603S, Lot # 16)
Validation	We only used commercial antibodies validated by the manufacturer and previously used in scientific publications. We also validated the antibodies in house through titration and use of positive and negative control whenever available

Eukaryotic cell lines

Policy information about cell lines and Sex and Gender in Research

Cell line source(s)	Cell lines were all from commercial sources as listed in Table S2
Authentication	Cell lines were authenticated by STR analysis as described in the Methods
Mycoplasma contamination	Cells were negative for Mycoplasma contamination
Commonly misidentified lines (See ICLAC register)	The KPL1 breast cancer cell line was determined to be a clonal derivative of the MCF7 breast cancer cell line. This cell line was used because it is a breast cancer cell line and shows genotypic and phenotypic differences compared to parental MCF7 cells (CLLE analysis and Saunus 2018; PMID: 28889351). The MCF7 orgin of KPL1 is mentioned in Table S2 of this manuscript.

Animals and other research organisms

Policy information about studies involving animals; ARRIVE guidelines recommended for reporting animal research, and Sex and Gender in Research

Laboratory animals	Female BALB/c nude mice (6-8 weeks old) were used in this study
Wild animals	The study did not involve wild animals
Reporting on sex	Only female mice were used because the in vivo experiments were focused on breast cancer
Field-collected samples	The study did not involve field-collected samples
Ethics oversight	No ethical approval or guidance required

Note that full information on the approval of the study protocol must also be provided in the manuscript.

Plants

Seed stocks	Not applicable. This study did not use plants
Novel plant genotypes	Not applicable. This study did not use plants
Authentication	Not applicable. This study did not use plants

Flow Cytometry

Plots

Confirm that:

☒ The axis labels state the marker and fluorochrome used (e.g. CD4-FITC).

☒ The axis scales are clearly visible. Include numbers along axes only for bottom left plot of group (a 'group' is an analysis of identical markers).

☒ All plots are contour plots with outliers or pseudocolor plots.

☒ A numerical value for number of cells or percentage (with statistics) is provided.

Methodology

Sample preparation	Cell lines grown and treated in 96 well plates were harvested by trypsinization and stained as described in detail in the Methods
Instrument	Novocyte 3005 (Agilent)
Software	NovoExpress 1.5.6 (Agilent)
Cell population abundance	This study tested the anti-proliferative and cytotoxic effects of anti-tumor drugs. As a consequence, treated samples had fewer cells than untreated samples. The number of cells analyzed was typically 1000-10000 in untreated samples. Since only cell lines were analyzed in this study, there were no issues with population purity.
Gating strategy	The gating strategy for the various analyses is described in the Methods and shown in Supplementary Figures 11, 12 , and 13. Generally, cells were first gated by forward and side scatter to exclude cell debris and to identify singlets. Live cells were gated by Zombie staining, which separates live and dead cells in clearly distinct populations. The live cell population was further analyzed for the metric of interest.

☒ Tick this box to confirm that a figure exemplifying the gating strategy is provided in the Supplementary Information.
